# The ‘Tommy Atkins’ mango genome reveals candidate genes for fruit quality

**DOI:** 10.1186/s12870-021-02858-1

**Published:** 2021-02-22

**Authors:** Mango Genome Consortium, Ian S. E. Bally, Aureliano Bombarely, Alan H. Chambers, Yuval Cohen, Natalie L. Dillon, David J. Innes, María A. Islas-Osuna, David N. Kuhn, Lukas A. Mueller, Ron Ophir, Aditi Rambani, Amir Sherman, Haidong Yan

**Affiliations:** 1 Department of Agriculture and Fisheries, Horticulture and Forestry Science, 28 Peters St, Mareeba, QLD 4880 Australia; 2 Department of Bioscience, University of Milan, Via Celoria 26, 20133 Milan, Italy; 3 School of Plants and Environmental Sciences, Virginia Tech, Ag Quad Lane, Blacksburg, VA 24061 USA; 4 Tropical Research and Education Center, Horticultural Sciences Department, University of Florida, 18905 SW 280th St, Homestead, FL 33031 USA; 5 Department of Fruit Tree Sciences, Volcani Research Center, Derech Hamacabim 68, P.O. Box 15159, 7528809 Rishon Le’Zion, Israel; 6 Department of Agriculture and Fisheries, Horticulture and Forestry Science, EcoSciences Precinct, 41 Boggo Rd, Dutton Park, QLD 4102 Australia; 7 Centro de Investigación en Alimentación y Desarrollo, A.C, Carretera Gustavo Enrique Astiazarán Rosas 46, Col. La Victoria, 83304 Hermosillo, Sonora, Mexico; 8 Subtropical Horticulture Research Station, USDA-ARS, 13601 Old Cutler Rd, Coral Gables, FL 33158 USA; 9 Boyce Thompson Institute, 533 Tower Road, Ithaca, NY 14853 USA

**Keywords:** *Mangifera indica*, fruit weight, QTL, de novo assembly

## Abstract

**Background:**

Mango, *Mangifera indica* L., an important tropical fruit crop, is grown for its sweet and aromatic fruits. Past improvement of this species has predominantly relied on chance seedlings derived from over 1000 cultivars in the Indian sub-continent with a large variation for fruit size, yield, biotic and abiotic stress resistance, and fruit quality among other traits. Historically, mango has been an orphan crop with very limited molecular information. Only recently have molecular and genomics-based analyses enabled the creation of linkage maps, transcriptomes, and diversity analysis of large collections. Additionally, the combined analysis of genomic and phenotypic information is poised to improve mango breeding efficiency.

**Results:**

This study sequenced, de novo assembled, analyzed, and annotated the genome of the monoembryonic mango cultivar ‘Tommy Atkins’. The draft genome sequence was generated using NRGene de-novo Magic on high molecular weight DNA of ‘Tommy Atkins’, supplemented by 10X Genomics long read sequencing to improve the initial assembly. A hybrid population between ‘Tommy Atkins’ x ‘Kensington Pride’ was used to generate phased haplotype chromosomes and a highly resolved phased SNP map. The final ‘Tommy Atkins’ genome assembly was a consensus sequence that included 20 pseudomolecules representing the 20 chromosomes of mango and included ~ 86% of the ~ 439 Mb haploid mango genome. Skim sequencing identified ~ 3.3 M SNPs using the ‘Tommy Atkins’ x ‘Kensington Pride’ mapping population. Repeat masking identified 26,616 genes with a median length of 3348 bp. A whole genome duplication analysis revealed an ancestral 65 MYA polyploidization event shared with *Anacardium occidentale*. Two regions, one on LG4 and one on LG7 containing 28 candidate genes, were associated with the commercially important fruit size characteristic in the mapping population.

**Conclusions:**

The availability of the complete ‘Tommy Atkins’ mango genome will aid global initiatives to study mango genetics.

**Supplementary Information:**

The online version contains supplementary material available at 10.1186/s12870-021-02858-1.

## Background

Mangoes are an important fruit crop grown in over 103 countries across the tropical and subtropical zones. The common mango is typically a large, tropical, and evergreen tree with an upright to spreading dense canopy that can reach up to 30 m in some climates if not pruned. Mango production is estimated to be over 50 million metric tons (Mt) per annum from an area of over 56.8 million hectares [1, 2]. India is by far the largest mango producer with 41.6% of world production (18 Mt) followed by China with 10% (4.5 Mt). The bulk of production is grown and consumed locally with only approximately 9.5 Mt exported due to the high local consumption in the countries of origin and the highly perishable nature of the fruit [1, 3].

Mango (*Mangifera indica* L.) belongs to the family Anacardiaceae. Based on morphological characters there are thought to be from 45 [4] to 69 [5] species within the *Mangifera* genus originating mainly in tropical Asia, with the area of highest diversity found in western Malesia [6]. The common mango, *M. indica,* was domesticated at least 4000 years ago, and further developed from an origin in the Assam Valley close to the western border of the Myanmar-Indochinese area in the Quaternary period and spread throughout the Indian subcontinent [6–8]. A further 26 species also have edible fruit, including *M. altissima*, *M. caesia*, *M. foetida*, *M. kemang*, *M. laurina*, *M. odorata*, *M. pajang* and *M. pentandra* being traditionally consumed in various Southeast Asian communities [9–11].

Although domestication and selection of mango varieties have occurred for thousands of years, the systematic breeding of mangoes is relatively recent, compared with many temperate tree fruit crops. Systematic mango breeding is a long term endeavor (up to 25 years) due to long juvenility, polyembryony, and very low fruit retention that reduce breeding efficiency and add time to the breeding generation cycle [12]. As a result, the general understanding of mango genetics and trait heritability has been limited. In more recent times, systematic breeding programs have aimed to develop varieties with production, consumer, and transportability traits more suited for national and international markets. Breeding mangoes with improved traits like reduced tree vigor, regular high yields, disease tolerance, long shelf life, optimal fruit size, shape, color, and high eating qualities are of primary interest to improve production efficiency and consumer demand [12, 13].

‘Tommy Atkins’ comes from a relatively recently developed group of cultivars that originated in Florida, USA, as chance seedlings in the early part of the twentieth century [14–16]. Their success is partly attributed to their relatively higher yields, large fruit size, strong blush color, lower vigor canopies, and adaptability across tropical and subtropical regions. This group originated from the high yielding monoembryonic cultivar ‘Mulgoba’ imported from India to the USA in 1910. An early seedling selection from ‘Mulgoba’ was named “Haden” which itself gave rise to the monoembryonic cultivars ‘Keitt’, ‘Kent’, and ‘Tommy Atkins’ that dominate international trade. Another cultivar, ‘Kensington Pride’, has dominated Australian production for the past century and is only now slowly being replaced by newer cultivars that generally have ‘Kensington Pride’ in their pedigree. The pre-Australian origin of ‘Kensington Pride’, prior to its introduction at Port Denison (now Bowen) between 1885 and 1889, is unknown. ‘Kensington Pride’ has a distinctive flavor and aroma not common in other Indian or Floridian cultivars. Its shape and red blush color suggest it has an Indian sub-continent origin, while its polyembryonic nature suggests a Southeast Asian origin. It has been suggested that ‘Kensington Pride’ is possibly a hybrid with Indian and Southeast Asian parentage [17].

The size of fruit on the earlier domesticated mango varieties was typically small as can be seen in older *M. indica* varieties and other species growing in north-east India, the Andaman Islands and throughout South East Asia [8]. Fruit size has been a priority breeding objective in mango, and selection over time has increased the average size of popular traded mango varieties up to 400 g [12, 18–21]. Fruit size has been estimated to have a high heritability [22]. In addition to fruit size, firmness, color, aroma production and stress response are quality characteristics of this climacteric fruit that need to be investigated at the genomic level to improve mango fruit quality. Short shelf life, high susceptibility to chilling injury and postharvest diseases are the major challenges that affect mango marketing [23]. Textural softening is a major quality attribute for consumer acceptance, and it is related to cell wall polysaccharides and their degrading enzymes [24]. Among the cell wall degrading enzymes that are relevant for mango softening (extensive pectin degradation) are exo-polygalacturonase, pectin methylesterase, (1–4)-beta-glucanase and beta-galactosidase [25].

Recent studies have improved our genetic and genomic information on mangoes including molecular analyses of germplasm collections [26–30], single-nucleotide polymorphism genotyping assays [31, 32], genetic maps with all 20 linkage groups [13, 33, 34], and transcriptomes [27–29, 32, 35–40]. All genetic and genomic information supports mango as a diploid with 40 chromosomes, which suggests the haploid number of chromosomes as 20. This agrees with the presence of 20 linkage groups. The haploid genome size is estimated at ~ 439 Mb [41]. In the absence of a homozygous cultivar, the mango cultivar ‘Tommy Atkins’ was selected for genome sequencing because it is one the most widely grown and internationally traded cultivars.

A high-quality genome can greatly aid mango breeders. Candidate gene homologs for important horticultural traits can be identified through combined “omics” approaches including comparative genomics with other tree crops. Single nucleotide polymorphism (SNP) markers for the candidate genes can be designed to screen hybrids at the seedling stage to identify associations between marker genotypes and horticultural traits. This will improve breeding efficiency and reduce the cost of selection by discarding inferior seedlings that do not carry favorable alleles. This report describes the creation of a chromosome-level genome for the primary commercial mango cultivar ‘Tommy Atkins’. The utility of the genome is shown by the identification of candidate genes for fruit size. The genome will serve as a resource for the global research community to advance the study of mango genetics and breeding.

## Results

### Genome sequencing, assembly, and annotation

The *M. indica* ‘Tommy Atkins’ mango genome was sequenced and de novo assembled using a combination of short reads, NRGene De-Novo Magic, 10X Genomics long read sequencing, and a phased SNP map. The result was a haploid sequence consensus assembly, TA4, with a total sequence length of 377 Mb in 571 scaffolds (Table 1). 87% of the assembly was anchored to 20 diploid pseudomolecules with an average length of 16.43 Mb (Supplementary Table 1). The TA4 assembly comprised 86% of the estimated size of the mango genome (~ 439 Mb) [41]. The overall GC content was 33.65%.

**Table 1:** Assembly statistics for the consensus diploid assembly of ‘Tommy Atkins’ (assembly version TA4)

Assembly statistics	Consensus diploid assembly TA4
Total scaffolds	571
Assembly size (bp)	377,281,443
Gaps size (bp)	8,765,473
Gaps %	2.32
N50 (bp)	7,734,592
N50 #sequences	16
N90 (bp)	1,660,372
N90 #sequences	20

The completeness of the gene space captured by the assembly was evaluated using two different approaches. First, BUSCO analysis indicated that 97.4% of the core genes were fully captured by this assembly. BUSCO analysis also identified 12.6% duplicated genes and 0.4% fragmented genes (Supplemental Table 2). Second, mapping three public RNA-Seq datasets from different tissues and conditions (SRP066658, SRP179820, SRP192932) delivered mapping rates of 92.57, 87.33, and 78.95%, respectively.

Repetitive elements represented 48% of the genome assembly (181,373,851 bp). Transposable elements (TE) class I were the most abundant elements accounting for the 34% of the genome (128,898,479 bp). As it is common in plant genomes, LTR/Copia and LTR/Gypsy were the most abundant TE Class I representing 13 and 14% of the assembly, respectively. TE Class II represented 12% of assembly (45,931,312 bp), and Helitrons represented 2% of the assembly (6,544,060 bp) (Supplemental Table 3). The dating of the insertion ages for the LTR/Copia and LTR/Gypsy revealed different ages of expansions for both groups. For the LTR/Copia there are two moderate expansions around 0.1 and 3.0 MYA, and for LTR/Copia there is a strong expansion around 1.7 MYA (Supplemental Figure 1).

26,616 gene models and 30,344 transcripts were predicted on the *M. indica* genome assembly TA4 using MAKER-P (Table 2). The gene annotation completeness was also evaluated with the BUSCO Embryophyta 10 dataset. 94.6% of the BUSCO genes were found in the *M. indica* gene model structural annotation. The percentage of duplicated genes was slightly higher than in the genomic sequences at 17.7%, and the percentage of fragmented BUSCO was also slightly higher at 1.7% (Supplemental Table 2). 89.0% of the transcripts were functionally annotated with at least one function derived from a BLAST homology search with the ARAPORT11, SwissProt, and NCBI NR databases or a protein domain search with InterproScan. The highest percentage of protein domains included pentatricopeptide (PPR) repeats, leucine rich repeats, and protein kinase domains (Supplemental Table 4).

**Table 2:** Annotation descriptive statistics. Results are shown for multiple descriptive statistics

Descriptive Statistic	Element Count	Mean size (bp)	Longest size (bp)	Total size (Mb)	Genome Percentage
Genes	26,616	4946	124,444	131.67	34.9
Transcripts	30,233	4903	124,444	130.52	34.6
CDSs	30,233	1446	20,694	38.54	10.2
Exons	213,768	255	7986	47.06	12.5
Five prime UTRs	19,115	207	3473	3.04	0.8
Three prime UTRs	20,006	344	4564	5.48	1.5
**Counting Statistics**	**Count**				
Transcripts with UTRs both sides	16,583				
Transcripts with UTR at least one side	22,538				
Single exon genes	1250				
Mean transcripts per gene	1.1				
Mean exons per transcripts	7.1				
Mean five prime UTR per transcript	0.6				
Mean three prime UTR per transcript	0.7				

### Evolutionary analysis of the mango genome

**Fig. 1:**
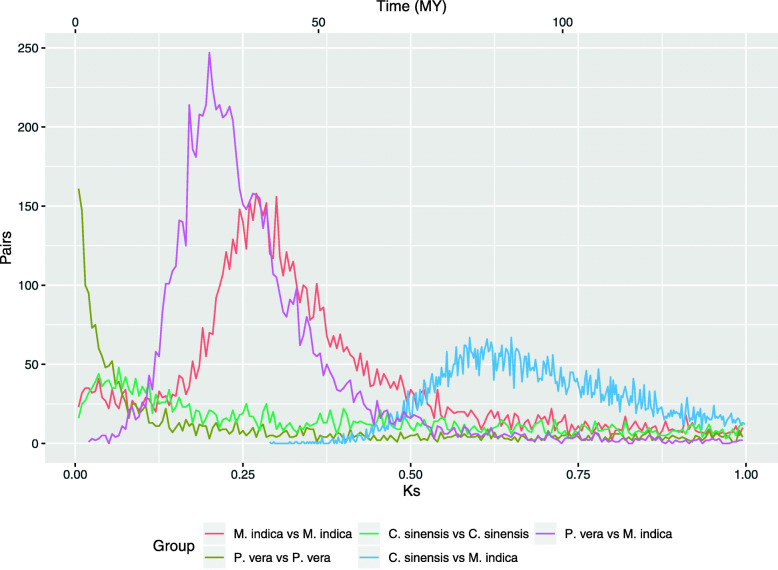
Comparison of whole genome duplication events among *P. vera*, *M. indica*, and *C. sinensis*

The mango genome was compared against itself and other selected genomes in order to elucidate part of the evolutionary history. *Citrus sinensis* (order Sapindales) was used as an outgroup species (different family, same order) to calibrate the divergence between Anacardiaceae and Rutaceae at 81 MYA [42]. A Ks distribution analysis of the coding sequences revealed that the mango genome had a whole genome duplication (WGD) dated at 65 MYA (Ks = 0.270). It is not shared with *Pistachia vera* from which it diverged 61 MYA (Ks = 0.200) in agreement with other phylogenetic studies (Fig. 1) [42]. It is possible that *P. vera* shared the same WGD event with *M. indica*, but it has been obscured by the collapsing of the homolog genes during the genome assembly. An alternative scenario is that the *Pistachia* ancestor diverged from the common ancestor with *Mangifera* a few million years before the WGD event. This alternative scenario is in agreement with some of the karyotypes in other members of the Anacardiaceae family. Some phylogenetic studies divide the family in three groups including A1, A2, and B [43]. Genera like *Mangifera*, *Anacardidium*, and *Semecarpus* in the group A1, have twenty or more pairs of chromosomes (2n = 2x = 40 [44], 2n = 2x = 42 [45], and 2n = 4x = 60 [46], respectively). Genera like *Pistachia*, *Toxicodendron*, and *Cotinus* from the group A2 have fifteen pairs of chromosomes (2n = 2x = 30) [47–49]. Under this scenario, an ancestor of the A group with fifteen pairs of chromosomes was derived in two ancestral species, A1 and A2. A2 maintained the same number of chromosomes while A1 had a WGD event duplicating the chromosomes to thirty pairs of chromosomes. In genus such as *Mangifera* and *Anacardidium*, they went through reduction in the number of chromosomes until twenty and twenty one pairs resulted, respectively.

**Fig. 2:**
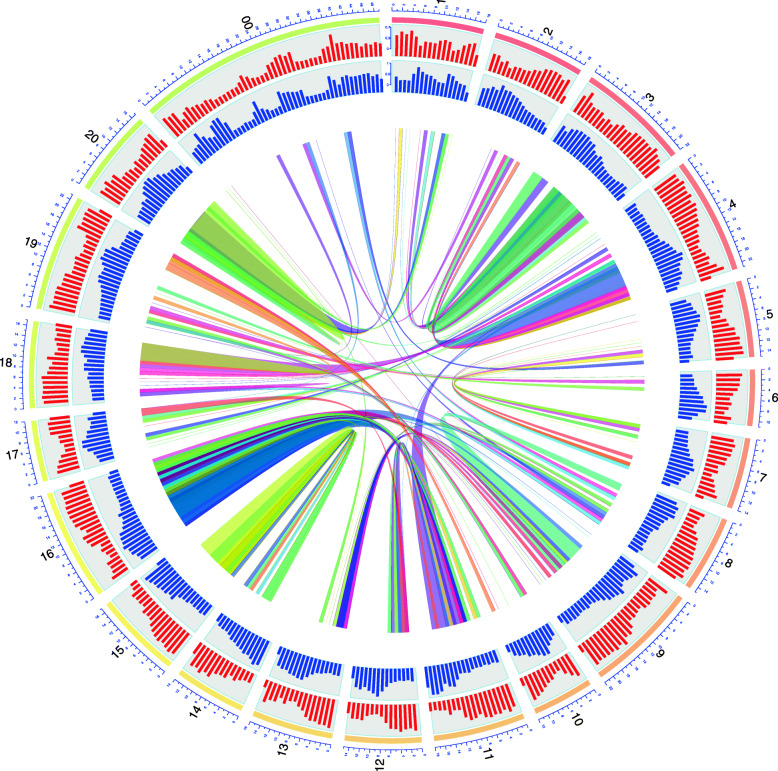
Circos plot of the *M. indica* cv. ‘Tommy Atkins’ genome with 20 pseudomolecules. External circles indicate the abundance of repeats (red) and gene density (blue). The internal circle depicts syntenic regions with > 10 genes with Ks values between 0.1 and 0.6 corresponding with whole genome duplication

**Fig. 3:**
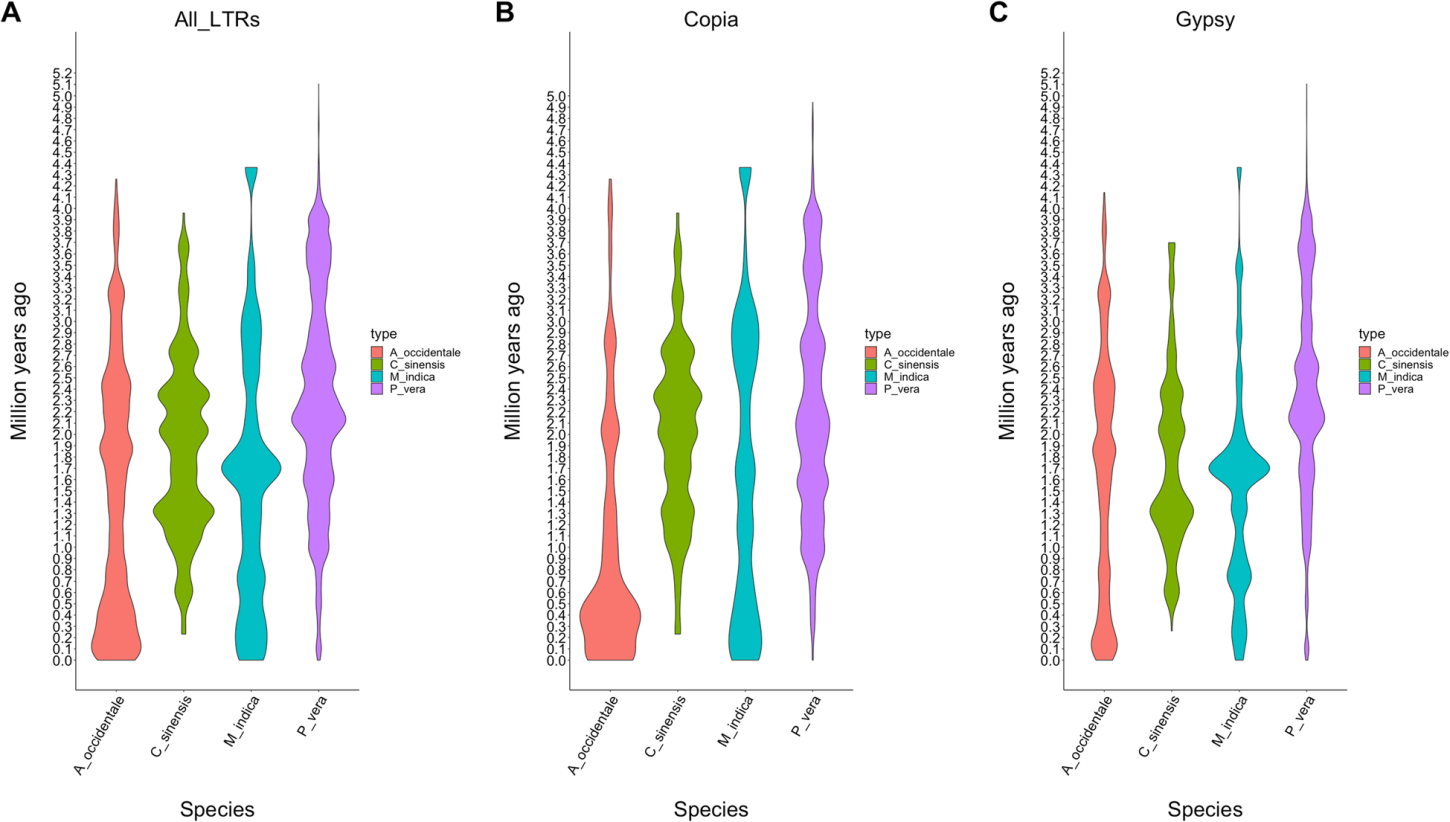
Insertion times for LTR elements for *C. sinensis* (red), *M. indica* (green), and *P. vera* (blue)

The analysis of the homolog gene pairs derived from the WGD showed synteny between the mango chromosomes (Fig. 2). For example, chromosomes 14 and 15 shared 518 gene pairs and chromosomes 11 and 16 shared 509 (Supplemental Table 5). The repetitive landscape was also compared among the three species analyzed for WGD. These three species showed similar levels of repetitive elements ranging from *C. sinensis* (39% of the genome assembly) to *P. vera* (66% of the genome assembly). The content of LTR/Copia are smaller than the content in LTR/Gypsy, although in the mango genome they are close to each other (13 and 14% respectively), compared with *C. sinensis* (10 and 14%) and *P. vera* (20 and 32%). The Class II TE was significantly higher in *M. indica* (12%) compared with the other three species (9% for *C. sinensis* and *P. vera*) (Supplementary Table 2). Although the LTR profiles are similar among these three species (Supplementary Figure 1), the insertion times for the last 5 MYA are different. *M. indica* and *C. sinensis* present a strong recent expansions of LTR/Gypsy 1.7 MYA and 1.3 MYA, respectively (Fig. 3).

### Comparison of ‘Tommy Atkins’ and ‘Kensington Pride’

Reads from mango cultivars ‘Tommy Atkins’ (monoembryonic) and ‘Kensington Pride’ (polyembryonic) were mapped onto the TA4 diploid consensus assembly to identify variants and effects using snpEff. For ‘Tommy Atkins’, a total of 5,394,956 variants comprised of 3,946,854 SNPs, 1,051,633 MNPs (multi-nucleotide polymorphisms), 138,977 insertions, 146,654 deletions, and 110,838 mixed variants were identified. The average variant rate for ‘Tommy Atkins’ was one variant every 69 bp. Similarly, the number of variants for ‘Kensington Pride’ was identified by mapping reads from the ‘Kensington Pride’ mango cultivar onto the TA4 assembly. The total number of variants was 9,030,142 comprised of 6,291,666 SNPs, 568,959 MNPs, 223,249 insertions, 245,632 deletions, and 700,636 mixed variants. ‘Kensington Pride’ has 1.67 times the number of variants of ‘Tommy Atkins’ and an average variants rate of one variant every 41 bp. Variant effects by impact, functional class, type, and region along with the full snpEff summary are reported in Supplemental Data S1 for ‘Tommy Atkins’ and Supplemental Data S2 for ‘Kensington Pride’.

### Mango gene family analysis

**Fig. 4:**
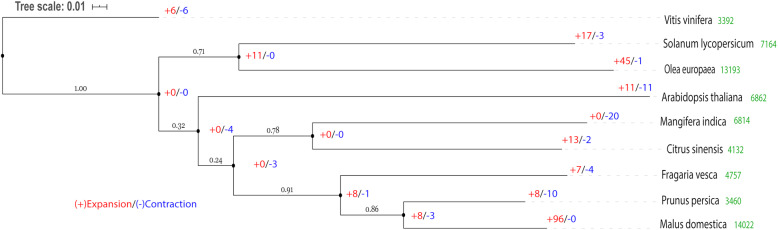
Species Tree inferance from All Genes (STAG) analysis of the mango genome. Branch support values indicate the proportion of gene trees with the same bipartition out of the total that were included in the analysis. Duplication events labeled at terminal branches were supported by occurrence in at least 50% of the descendant species. Significantly contracting and expanding gene families with *p* value < 0.05 using CAFÉ are denoted by blue and red color respectively

The proteomes of eight additional plant species were used for a comparative study with mango including *Arabidopsis*, four species of climacteric fruits (*Solanum lycopersicum*, *Malus domestica*, *Prunus persica*), and four species of non climacteric fruits (*Vitis vinifera*, *Citrus sinensis*, *Fragaria vesca*, *Olea europaea*). There were 17,382 orthogroups identified containing 219,431 genes (75.3% of total input) using Orthofinder [50]. Orthogroups containing all species were used to infer the species tree, 914 of these consisted entirely of single copy genes. Gene duplication events are designated by the number at the end of terminal branches of the species tree (Fig. 4). Shown are duplications for which both copies were retained in at least 50% of the descendant species. There were no significantly expanding gene families in mango, but gene families involved in fruit ripening like plant invertase/pectin methylesterase inhibitor were significantly contracted (Table 3).

**Table 3:** List of rapidly evolving gene families in mango with *p* value < 0.05 determined by CAFE

Gene Family (PFAM ID)	Function	Contraction (−) Expansions (+)
PF04043	Plant invertase/pectin methylesterase inhibitor	−22*
PF05617	Prolamin-like	−14*
PF00295	Glycosyl hydrolases family 28	−13*
PF02798	Glutathione S-transferase, N-terminal domain	−10*
PF01565	FAD binding domain	−10*
PF03478	Protein of unknown function (DUF295)	−9*
PF05938	Plant self-incompatibility protein S1	−15*
PF00891	O-methyltransferase domain	−13*
PF00232	Glycosyl hydrolase family 1	−10*
PF00954	S-locus glycoprotein domain	−15*
PF12819	Malectin-like domain	−11*
PF03492	SAM dependent carboxyl methyltransferase	−9*
PF13947	Wall-associated receptor kinase galacturonan-binding	−11*
PF07859	alpha/beta hydrolase fold	−13*
PF03018	Dirigent-like protein	−10*
PF05056	Protein of unknown function (DUF674)	−9*
PF14226	non-haem dioxygenase in morphine synthesis N-terminal	−9*
PF14291	Domain of unknown function (DUF4371)	−7*
PF09331	Domain of unknown function (DUF1985)	−5*
PF11820	Protein of unknown function (DUF3339)	−5*

### Association of SNPs and fruit weight using the mango genome.

**Fig. 5:**
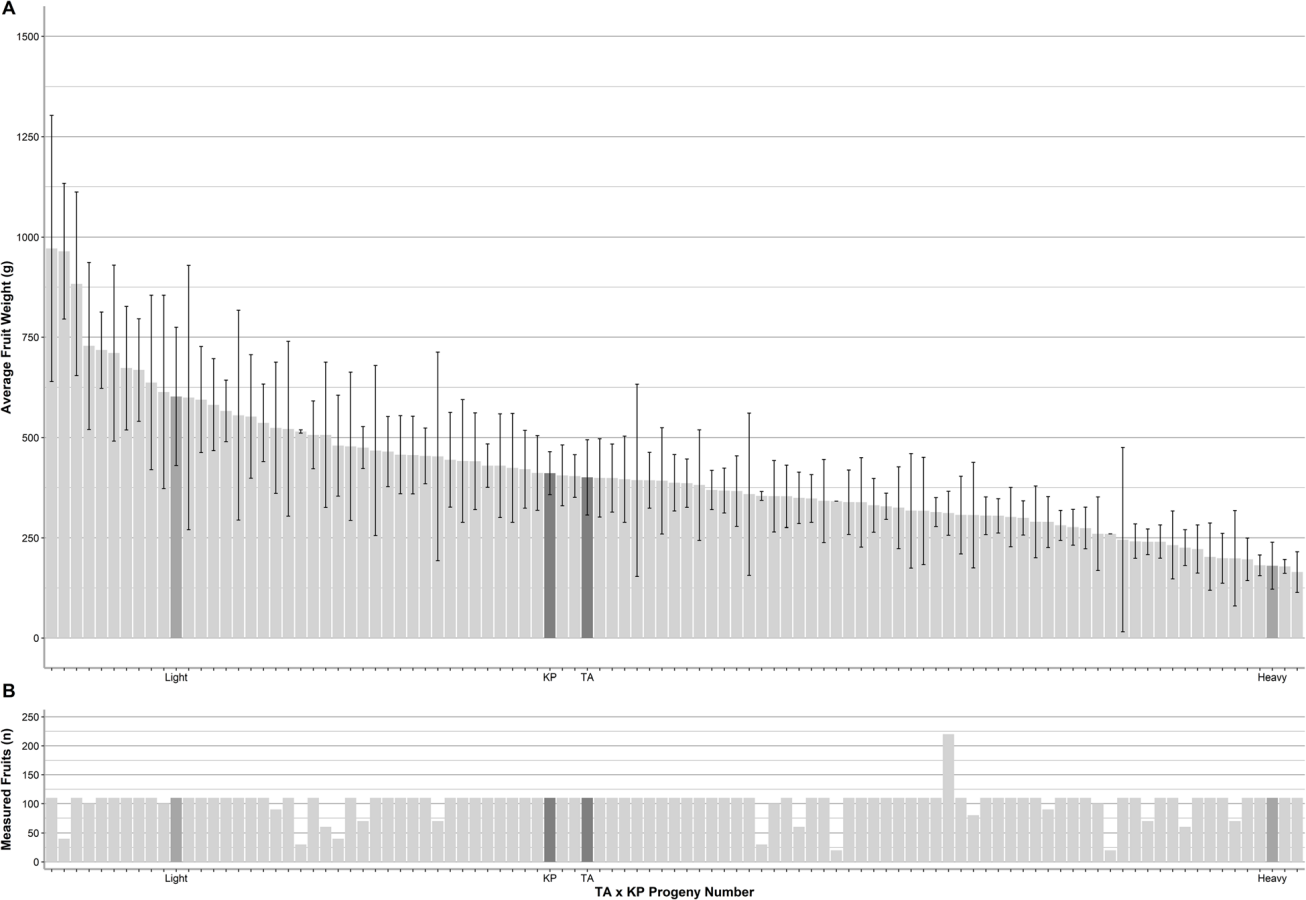
Fruit weight phenotypic data for the ‘Tommy Atkins’ x ‘Kensington Pride’ progeny. **a** Average fruit weight for ‘Tommy Atkins’, ‘Kensington Pride’, and 99 progeny in the hybrid population. Dark grey bars represent the two parents ‘Tommy Atkins’ and ‘Kensington Pride’. Medium grey bars represent the light and the heavy fruit presented in Fig. 6. Error bars represent the standard deviation. **b** The total number of fruit used to develop the mean fruit weight for each parent and progeny

**Fig. 6:**
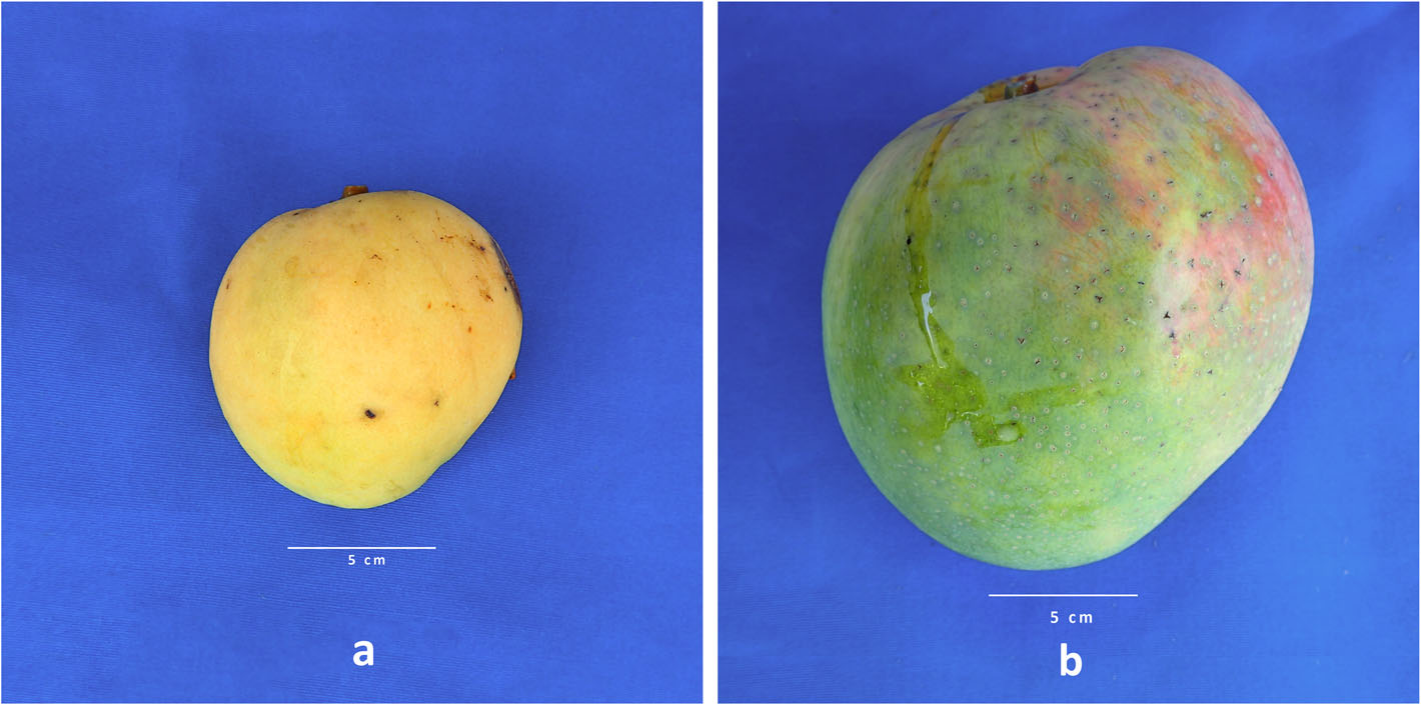
Photographs of two progeny from the ‘Tommy Atkins’ x ‘Kensington Pride’ mapping population. **a** Accession 7013 has some of the lightest fruit with an average fruit weight of 210 g, a diplotype of TA2KP1 in the LG4 region and TA1KP1 in the LG7 region. **b** Accession 9020 has some of the heaviest fruit with an average fruit weight of 867 g, a diplotype of TA2KP2 in the LG4 region and TA2KP1 in the LG7 region

The availability of a genome enables genetic dissection of important traits. While ‘Tommy Atkins’ and ‘Kensington Pride’ are similar for many similar traits (Table 4), their progeny showed high variation for fruit weight (Figs. 5 and 6). Average fruit weight for ‘Tommy Atkins’ and ‘Kensington Pride’ parents of the hybrid population was 400 g and 410 g, respectively, with hybrid progeny ranging from 165 g and 971 g. Association of SNPs with fruit weight identified two QTL regions at estimated *p*-values of 0.001. One region was identified on LG4 from bp position 8,275,233 to 8,495,231, and one on LG7 from bp positions 3,831,615 to 3,914,160.

**Table 4:** Phenotypic and morphological characteristics between ‘Tommy Atkins’ and ‘Kensington Pride’ [48, 49]

Phenotypic and morphological trait	‘Tommy Atkins’	‘Kensington Pride’
Embryony	Monoembryonic	Polyembryonic
Fruit weight (g)	400.1 g	410.9 g
Fruit dimensions (LxWxD in mm)	77 × 36 × 20	109 × 92 × 42
Fruit shape	Ovate round	Ovate
% flesh recovery	77%	79%
Canopy habit	Upright	Spreading
Yield	High	Medium
Canopy openness	Medium to open	Dense
Tree vigor	Semi dwarf	Vigorous
Seasonality	Mid to late	Early
Fruit blush color at ripe	Yellow with a strong burgundy blush all over	Yellow with pink blush up to 45% of skin
Fibrous flesh level	Medium	Low
Firmness at ripe	Firm	Soft
Fruit retained on panicle	One to three	One

### Genes in the fruit weight QTL regions

Using the position boundaries of the QTL regions, a total of 28 genes were found in the LG4 region and seven genes in the LG7 QTL region (Table 5). All genes in the region are reported without filtering based on their potential effect on fruit weight.

**Table 5:** Genes in the fruit weight QTL regions. These regions were associated with fruit weight in the ‘Tommy Atkins’ x ‘Kensington Pride’ population

Linkage Group	Base pair position	Gene annotation
4	8,276,995	MVP1: Inactive GDSL esterase/lipase-like protein 25 (*Arabidopsis thaliana*)
4	8,277,387	GLIP5: GDSL esterase/lipase 5 (*Arabidopsis thaliana*)
4	8,281,294	B120: G-type lectin S-receptor-like serine/threonine-protein kinase B120 (*Arabidopsis thaliana*)
4	8,283,451	Protein of unknown function
4	8,284,126	DRG1: Developmentally-regulated G-protein 1 (*Arabidopsis thaliana*)
4	8,302,340	SARED1: Sanguinarine reductase (*Eschscholzia californica*)
4	8,307,120	EFL4: Protein ELF4-LIKE 4 (*Arabidopsis thaliana*)
4	8,314,410	TAF12B: Transcription initiation factor TFIID subunit 12b (*Arabidopsis thaliana*)
4	8,326,478	C1: Anthocyanin regulatory C1 protein (*Zea mays*)
4	8,346,564	ATG12: Ubiquitin-like protein ATG12 (*Medicago truncatula*)
4	8,351,941	WER: Transcription factor WER (*Arabidopsis thaliana*)
4	8,360,040	AIL1: AP2-like ethylene-responsive transcription factor AIL1 (*Arabidopsis thaliana*)
4	8,374,018	GRF5: Growth-regulating factor 5 (*Arabidopsis thaliana*)
4	8,380,107	GLP1: Germin-like protein subfamily 3 member 1 (*Arabidopsis thaliana*)
4	8,382,500	SPL2: E3 ubiquitin-protein ligase SPL2 (*Arabidopsis thaliana*)
4	8,388,401	TAF9: Transcription initiation factor TFIID subunit 9 (*Arabidopsis thaliana*)
4	8,390,867	RSH2: Probable GTP diphosphokinase RSH2%2C chloroplastic (*Arabidopsis thaliana*)
4	8,402,524	PSD: Exportin-T (*Arabidopsis thaliana*)
4	8,408,800	Cabp1: Calcium-binding protein 1 (*Rattus norvegicus*)
4	8,418,968	RIPK: Serine/threonine-protein kinase RIPK (*Arabidopsis thaliana*)
4	8,430,804	CYP75B1: Flavonoid 3′-monooxygenase (*Arabidopsis thaliana*)
4	8,438,631	LOX3.1: Linoleate 13S-lipoxygenase 3–1%2C chloroplastic (*Solanum tuberosum*)
4	8,458,547	GG3: Guanine nucleotide-binding protein subunit gamma 3 (*Arabidopsis thaliana*)
4	8,462,119	CCX4: Cation/calcium exchanger 4 (*Arabidopsis thaliana*)
4	8,471,534	ALDH7B4: Aldehyde dehydrogenase family 7 member B4 (*Arabidopsis thaliana*)
4	8,477,434	Protein of unknown function
4	8,487,623	DAGLA: Sn1-specific diacylglycerol lipase alpha (*Homo sapiens*)
4	8,492,535	RPL9: 60S ribosomal protein L9 (*Pisum sativum*)
7	3,833,896	Protein of unknown function
7	3,840,639	Coq9: Ubiquinone biosynthesis protein COQ9%2C mitochondrial (*Drosophila melanogaster*)
7	3,852,329	TIFY10A: Protein TIFY 10A (*Arabidopsis thaliana*)
7	3,866,715	MARD1: Protein MARD1 (*Arabidopsis thaliana*)
7	3,882,853	BGAL15: Beta-galactosidase 15 (*Arabidopsis thaliana*)
7	3,888,807	ERF017: Ethylene-responsive transcription factor ERF017 (Arabidopsis thaliana)
7	3,890,361	CPN20: 20 kDa chaperonin%2C chloroplastic (*Arabidopsis thaliana*)
7	3,892,877	ARF19: Auxin response factor 19 (*Arabidopsis thaliana*)

### Association of haplotype to fruit weight

The haplotype of each parent contributing the minor allele for each SNP associated with fruit weight was identified using the phased genetic map of ‘Tommy Atkins’ x ‘Kensington Pride’. The genotypes of each individual in the ‘Tommy Atkins’ x ‘Kensington Pride’ population for all SNPs was inferred from the phased genetic map and haplotype data. PLINK analysis identified SNPs in the QTL regions and their associated haplotype. The four possible diplotypes (haplotype from each parent) were determined (TA1KP1, TA1KP2, TA2KP1, TA2KP2) for each individual in the QTL region. Only two haplotypes (one from ‘Tommy Atkins’ and one from ‘Kensington Pride’ were observed in the QTL region for the majority of individuals. Three putative instances of recombination were identified in the LG4 region and one in the LG7 region for the population and these individuals were not included in the respective diplotype analysis of the region. An ANOVA analysis of the fruit weight data using the diplotypes as groups showed a significant difference among groups for both LG4 and LG7. The post hoc Tukey’s multiple comparison of means test showed a significant difference between diplotypes TA2KP1 and TA2KP2 for LG 4 and no significant paired group difference for LG 7 (Supplemental data S3).

### Web resources for mango genomics

An online genome resource was created to support mango research (https://mangobase.org). Mangobase contains a genome browser that displays the mango reference sequence with gene sequences, annotations, repeat locations, SNP tracks, and other data. Other tools including BLAST are available for use with datasets related to the genome sequence, CDS, and protein datasets. Genetic maps can also be viewed on mangobase.org. The system is based on the SGN platform (https://solgenomics.net/) [51], which includes many other features including a community curation system for genetic loci and advanced data management for field and genotyping experiments. All data can be downloaded as complete datasets from an FTP site at ftp://ftp.mangobase.org/.

## Discussion

Generating an annotated genome assembly for a tree crop like mango provides an essential genetic and genomic tool for improvement through plant breeding. The final ‘Tommy Atkins’ diploid consensus assembly, TA4, consisted of 20 pseudomolecules that were congruent with the two available genetic maps of mango. A phased map made by skim sequencing of 87 individuals of the ‘Tommy Atkins’ x ‘Kensington Pride’ hybrid population identified ~ 3 M SNP variants with known nucleotide positions in the genome. The result is a publicly available resource for the mango community, and for those interested in comparative genomics. Advances in sequencing and data analysis are enabling scientific discovery even for highly valued, yet historically under researched, species like mango.

Recently, the genome sequence of a mango cultivar ‘Alphonso’ was published [52]. The methods of sequencing, assembly, and annotation of the two genomes are significantly different. However, both genome assemblies used the same published map to finalize the assembly [33]. Whereas the ‘Tommy Atkins’ genome used the same linkage group numbers as the published mango map to identify pseudomolecules, the ‘Alphonso’ publication did not. The ‘Alphonso’ genome instead supplied the linkage group to which each pseudomolecule corresponded. Using this information, a table was generated to show the correspondence of the two assemblies by pseudomolecule (Supplemental Table S8). Lengths of each pseudomolecule were generally similar with the exception of an ~ 7 Mb larger pseudomolecule 19 in the ‘Alphonso’ assembly. The quality measures for both assemblies (e.g. BUSCO analysis) were also similar. A significant difference in the annotation of the two genomes included 41,251 predicted protein-coding genes in ‘Alphonso’ versus 26,616 protein-coding genes in ‘Tommy Atkins’. The ‘Alfonso’ repetitive sequence content was reported to be 40.5% compared to 48% for ‘Tommy Atkins’ genome.

In general, major differences in gene family analysis results between the ‘Alphonso’ mango genome and ours arise due to number of differences in input data and focus of our analysis. There were a total 41,251 protein coding genes annotated for ‘Alphonso’ in comparison to the 26,616 high quality genes in the ‘Tommy Atkins’ genome. This difference led to a reduced number of valid gene families used as input for Café analysis for the ‘Tommy Atkins’ genome compared ‘Alphonso’ (3281 and 3791, respectively). Additionally, the parameters invoked for the gene family analysis for ‘Tommy Atkins’ were very strict when eliminating possible transposable elements and were more conservative when including any gene families represented by more than 100 genes. For example, several disease resistance gene families (eg. NB-LRR) can be misrepresented by TE gene families inflating actual numbers and this can impact gene family results. Additionally, instead of using gene families automatically created by Orthofinder based on a reciprocal blast, manually consolidated counts based on shared pfam domain were used in this study. Finally, the focus of the current study was to compare differences between climacteric and non climacteric fruits and removing inflated gene families made the analysis in this study more accurate at identifying these differences. These differences may have resulted in contrasting results for assigning expanding versus contracting gene family results.

Using the renumbered pseudomolecules, Circos plots for both genomes were compared and generally found to be congruent. A good example is the synteny between pseudomolecules 14 and 15 for ‘Tommy Atkins’ and the corresponding Min11 and Min19 for ‘Alphonso’. The methods for generating the inferred phylogenetic tree and frequency distributions for synonymous substitutions to allow estimation of occurrence of whole genome duplication were different for ‘Alphonso’ and ‘Tommy Atkins’. Considering only data for the commonly included genomes, there were no significant differences in the inferred phylogenetic tree or in the estimation of the whole genome duplication event for mango. The analysis of expansion or contraction of gene families after the whole genome duplication event for mango gave different results for ‘Alphonso’ where the majority of gene families showed expansion in comparison to other genomes and ‘Tommy Atkins’ where gene families exhibited contraction in comparison to other genomes as described below.

Comparative genomic analyses between mango and fruit bearing species (climacteric and non-climacteric) revealed that there were no significantly expanded gene families in mango, only contractions. The pectin invertase/pectin methylesterase inhibitor gene family underwent the greatest contraction in the mango genome. Pectin invertase/pectin methylesterase inhibitor proteins are known to be involved in fruit ripening and softening. Other gene families that were contracted are glycosyl hydrolases family 28 (GH28) that includes polygalacturonases (endo, exo, and rhamno), glycosyl hydrolases family 1 (GH1) that includes beta-galactosidase and (1–4)-beta-glucanase and other proteins and unknown gene products (Table 3). Pectin methylesterase inhibitors (PMEI) are large multigene families in eudicots and modulate PME activity and the degree of methyl-esterification during fruit ripening [53, 54]. In Arabidopsis, 71 putative PMEI genes were identified [55], and 97 in *Brassica rapa* [56], while in the mango 18 PMEIs were identified. This family contraction could be in part related to the rapid loss of firmness (early softening related to pectin degradation) happening in mango fruits [37] due to reduced inhibition of PME. Upon PME action, polygalacturonases (PG) continue pectin hydrolysis; PG is one of the largest hydrolase families in plants, eudicots like *A. thaliana*, *Brassica rapa*, *Solanum tuberosum*, *Solanum lycopersicum*, *Populus trichocarpa*, *Glycine max*, *Citrullus lanatus* and *Cucumis sativus* have 68, 99, 49, 54, 76, 98, 62 and 53 genes, respectively [57]. However, in the mango genome 48 PGs were identified [58]; previously, only 17 PG genes were identified in the transcriptome of mango cv. Kent, and 9 of them were differentially expressed during ripening [36, 58]. Even though the PG family was contracted in the mango genome compared to other reference plants, seven of them are exo-PGs which are related to pectin modification and softening in mango fruit [25]. Thirty PMEs and twenty five beta-galactosidases were also identified in the ‘Tommy Atkins’ mango genome and are also cell-wall remodeling enzymes important for softening [25].

The availability of the ‘Tommy Atkins’ genome will enable greater insights from previous genomics research in mango. For example, a previous study identified transcripts for proteins involved in metabolic pathways related to mango fruit ripening and fruit quality [36]. Mapping these reads to the genome will facilitate additional in-depth analyses of transcripts related to polygalacturonases, cell wall proteins and enzymes, metabolism, ethylene biosynthesis and signaling, sucrose and carotenoid metabolic processes, and polysaccharide catabolic processes not only for coding sequences but also for regulatory regions in order to uncover the gene products that regulate gene expression.

We demonstrated the utility of the genome assembly and the ‘Tommy Atkins’ x ‘Kensington Pride’ map by analyzing average fruit weight phenotypic data. This is a highly valuable trait for both breeders and growers. Fruit weight of the parents of the mapping population was almost identical (~ 400 g), whereas fruit weight of the hybrids ranged from ~ 165 g to ~ 965 g (Table 4, Figs. 5 and 6), a strong example of transgressive segregation most likely arising from highly heterozygous parents. We were able to associate the fruit weight trait with two regions one on LG4 and one on LG7 with significant associations (*p*-value <= 0.001). Candidate genes in the LG4 region included the E3 gene encoding ubiquitin protein ligase. This same gene (Prupe.6G045900 E3 ubiquitin-protein ligase) was found in the QTL for fruit weight on chromosome 6 in peach (*Prunus persea*) [59]. Expression studies in peach showed a five fold increase in expression of Prupe.6Go45900 over the period of fruit development, the second largest increase in expression of the 19 candidate genes from the fruit weight QTL analyzed.

Mapping populations from controlled crosses are not common in mango due to the high level of technical proficiency required to create them. Most tree breeding populations are developed from open pollinated maternal parents of known commercial value and genetic screening to identify the paternal parent and generate a hybrid population from two known parents. In the ‘Tommy Atkins’ x ‘Kensington Pride’ population, both parents are valuable commercial cultivars and selections from their progeny have the potential to become commercial varieties. Both parents are highly heterozygous as commercial fruit trees are selected from seed of open pollinated maternal trees for favorable horticultural traits and subsequently vegetatively propagated. In general for trees, the F1 population studied is small compared to annual crops and development of maps and association of traits requires a pseudo-test cross approach (Grattapaglia and Sederoff, 1994). Our genetic recombination map with defined haplotypes for each SNP is unique to our mango mapping population. Thus, our analysis of the association of diplotype with fruit weight in the two QTL regions is also unique. It enables us to develop strategies that will improve the efficiency of identifying progeny with optimal commercially suitable fruit size from open pollinated progeny of either parent.

Associating fruit weight with particular diplotypes in the QTL regions implicated the LG4 region as a significant indicator of fruit weight. In the LG4 QTL region, diplotype TA2KP1 had the lowest average fruit weight (329 g) and TA2KP2 had the highest average fruit weight (499 g). As the TA2 haplotype is present in both the largest and smallest fruit, the major effect on fruit size is due to the KP parent with the KP1 haplotype decreasing fruit weight and KP2 haplotype increasing fruit weight. No significant distinction between diplotypes in the LG7 QTL was observed, but the two highest average fruit weights were for TA2KP1 (450 g) and TA2KP2 (468 g) while the two diplotypes TA1KP1 and TA1KP2 were almost identical, 352 and 359 g respectively. Thus, at the LG7 QTL, the TA parent has the greatest effect with TA1 decreasing fruit weight and TA2 increasing fruit weight. Diplotypes for each QTL region can be confidently predicted with 24 SNPs. Thus, ‘Tommy Atkins’ open pollinated progeny may be screened at the seedling stage for the presence of the TA2 haplotype at the LG7 QTL region. ‘Kensington Pride’ open pollinated progeny may be screened for the presence of KP2 at the LG4 QTL region. Identification of QTL for other horticultural traits is in progress and could lead to a suite of markers for advantageous haplotypes that could further improve selection efficiency at the seedling stage in open pollinated progeny of ‘Tommy Atkins’ and ‘Kensington Pride’.

## Conclusions

The Mango Genome Consortium successfully developed a mango genome for the most commercially important cultivar, ‘Tommy Atkins’, as a step towards a global and integrated initiative to study mango genetics. The sequencing of the ‘Tommy Atkins’ genome proved to be useful in identifying QTLs, genes, and diplotypes associated with fruit weight. We anticipate that the availability of the ‘Tommy Atkins’ genome and related resources at mangobase.org will lead to additional discoveries in the future.

## Methods

### The aim, design, and setting of the study

The aim of this study was to create a high-quality mango genome and demonstrate its utility using fruit weight as an example for trait dissection.

### Fruit weight measurements

Fruit weight was measured in fully mature fruit at harvest on a sample of ten randomly picked fruit from the ‘Tommy Atkins’ x ‘Kensington Pride’ parents and each of the 99 individual progeny within the ‘Tommy Atkins’ x ‘Kensington Pride’ hybrid population. The measurements were repeated over 5 years between 2007 and 2012.

### Plant materials and DNA extraction

The ‘Tommy Atkins’ mango cultivar was curated and maintained at the USDA Subtropical Horticultural Research Station in Miami, Florida, USA. A hybrid population from the cultivar ‘Tommy Atkins’ (TA, maternal parent) and cultivar ‘Kensington Pride’ (KP, paternal parent) consisting of 104 individuals was generated by hand pollination and maintained at the Department of Agriculture and Fisheries, Mareeba, Australia [60]. High molecular weight DNA was isolated from ‘Tommy Atkins’ leaf material by lysis of isolated nuclei. DNA from ‘Tommy Atkins’, ‘Kensington Pride’, and ‘Tommy Atkins’ x ‘Kensington Pride’ hybrids was isolated from leaf material using a Mag-Bind Plant DNA DS 96 Kit (Omega, M1130–01) according to manufacturer’s protocol with slight modifications to minimize degradation. Forty 3 mm leaf punches (about 40 mg) were ground once in CSPL extraction buffer and proteinase K on a Genogrinder 2000 at 1750 RPM for 2 min. After a 30 min incubation at 65 °C, samples were centrifuged at 4000 x g for 15 min and 500 ul of the lysate was transferred to a new 96 deep well plate. All remaining steps were performed on a Hamilton Microlab STARlet liquid handling robot according to Omega manufacturer protocol with all mixes performed by vortex instead of aspiration, and the final elution transferred manually.

### Genome sequencing, assembly, and annotation

‘Tommy Atkins’ high molecular weight DNA was subjected to library construction, sequencing, and assembly at NRGene (Israel). High molecular weight DNA quality was verified by pulsed-field gel electrophoresis. DNA fragments longer than 50 Kb were isolated to construct a Gemcode library using the Chromium instrument (10X Genomics, Pleasanton, CA). This library was sequenced on HiSeqX platform to produce 2 × 150 bp reads. Five size-selected genomic DNA libraries ranging from 470 bp to 10 Kb were constructed and two shotgun libraries were made with size selection of ~ 470 bp with no PCR amplification. This fragment size was designed to produce a sequencing overlap of the fragments on the Hiseq2500 v2 Rapid mode as 2 × 265 bp, thus creating an opportunity to produce ‘stitched’ reads of approximately 265 bp to 520 bp in length. The genomic library of 800 bp DNA fragment sizes was prepared using the TruSeq DNA Sample Preparation Kit version 2 with no PCR amplification according to the manufacturer’s protocol (Illumina, San Diego, CA). To increase sequence diversity and genome coverage, three separate MP libraries were constructed with 2–5 Kb, 5–7 Kb and 7–10 Kb jumps using the Illumina Nextera Mate-Pair Sample Preparation Kit (Illumina, San Diego, CA). The 800 bp shotgun library was sequenced on an Illumina HiSeq2500 as 2 × 160 bp reads (using the v4 illumina chemistry) while the MP libraries were sequenced on HiSeq4000 as 2X150 bp reads. For the 10x Chromium library, PE and MP libraries construction and sequencing were conducted at the Roy J. Carver Biotechnology Center, University of Illinois at Urbana-Champaign. 10X Chromium library construction and sequencing were conducted at HudsonAlpha Institute for Biotechnology, Huntsville, Alabama.

The newly assembled scaffolds were ordered into linkage groups using the high density preliminary maps created from the ‘Tommy Atkins’ x ‘Kensington Pride’ mapping population. The assembly was further improved using data from 10X Genomics sequencing (10X Genomics, Huntsville, AL). The consensus assembly (TA4) was generated and reduced to 20 pseudomolecules (linkage groups) plus unassembled scaffolds. The assembly was validated and revised by comparison to SNP order in two mango SNP maps [13, 33].

Genome assembly was conducted using DeNovoMAGIC™ software platform (NRGene, Nes Ziona, Israel). This is a DeBruijn-graph-based assembler, designed to efficiently extract the underlying information in the raw reads to solve the complexity of the DeBruijn graph due to genome polyploidy, heterozygosity and repetitiveness. This task is accomplished using accurate-reads-based traveling in the graph that iteratively connected consecutive phased contigs over local repeats to generate long phased scaffolds [61–65]. The additional raw Chromium 10X data was utilized to phase polyploidy/heterozygosity, support scaffolds validation and further elongation of the phased scaffolds. Heterozygous genome assembly using DeNovoMAGIC™ result in 2 assembly versions: Phased and Un-Phased.

For read pre-processing, PCR duplicates, illumina adaptors, and Nextera linkers (for MP libraries) were removed. The PE 450 bp 2 × 265 bp library overlapping reads were merged with minimal required overlap of 10 bp to create the stitched reads. Following pre-processing, merged PE reads were scanned to detect and filter reads with putative sequencing error (contain a sub-sequence that does not reappear several times in other reads). Contig assembly consisted of building a De Bruijn graph (kmer = 127 bp) of contigs from the all PE & MP reads. Next, PE reads were used to find reliable paths in the graph between contigs for repeat resolving and contigs extension. 10x barcoded reads were mapped to contigs ensure that adjacent contigs were connected only in case there is an evidence that those contigs originate from a single stretch of genomic sequence (reads from the same two or more barcodes were mapped to both contigs).

For split phased/un-phased assembly processes, two parallel assemblies took place to complete the phased and un-phased assembly result. The phased assembly process utilizes the complete set of contigs. In the un-phased assembly process, the homologous contigs are identified and one of the homologous is filtered out, leaving a subset of the homozygous and one of the homologous contigs in heterozygous regions. The linking information of both homologous contigs is kept through the assembly process of the un-phased assembly, usually enabling longer un-phased scaffolds.

For scaffold assembly, all the following steps were done in parallel for both the phased and un-phased assemblies. Contigs were linked into scaffolds with PE and MP information, estimating gaps between the contigs according to the distance of PE and MP links. In addition, 10x data was used to validate and support correct phasing during scaffolding. A final gap filling step used PE and MP links and De Bruijn graph information to detect a unique path connecting the gap edges. 10x barcoded reads were mapped to the assembled scaffolds and clusters of reads with the same barcode mapped to adjacent contigs in the scaffolds were identified to be part of a single long molecule. Next, each scaffold was scanned with a 20 kb length window to ensure that the number of distinct clusters that cover the entire window (indicating a support for this 20 kb connection by several long molecules) was statistically significant with respect to the number of clusters that span the left and the right edge of the window. In case where a potential scaffold assembly error was detected the scaffold was broken at the two edges of the suspicious 20 kb window. Finally, the barcodes that were mapped to the scaffold edges were compared (first and last 20 kb sequences) to generate a scaffolds graph with a link connecting two scaffolds with more than two common barcodes. Linear scaffolds paths in the scaffolds graph were composed into the final scaffolds output of the assembly.

### Phased recombination genetic map

The assembly of the TA4 scaffolds (including the 10X Genomics data) produces a partially phased assembly in the sense that each scaffold is originating from a single haplotype. To group these phased scaffolds into their haplotypic groups (e.g. find the linkage between scaffolds and place them in the same LG), a phased recombination genetic map was produced.

Illumina sequencing (5x coverage) of DNA from ‘Tommy Atkins’, ‘Kensington Pride’, and each of the ‘Tommy Atkins’ x ‘Kensington Pride’ hybrids was conducted by NRGene. Unique heterozygous SNPs from each of the parental lines of the cross were identified. All SNPs were homozygous in one parent and heterozygous in the other which allowed identification of the parental haplotype in each region of the map and the recombination points for each individual. Parental maps were generated by NRGene using a pseudo-testcross approach as described in (Grattapaglia and Sederoff, 1994). SNPs were mapped to the partially phased scaffolds of the TA4 assembly to identify their haplotype origin and location. SNPs were named by their nucleotide position on a pseudomolecule in the final TA4 assembly. A phased SNP map of the ‘Tommy Atkins’ x ‘Kensington Pride’ population with 20 linkage groups was created with ~ 3.3 M SNPs. The genotype and haplotype of each of the hybrid individuals at each SNP was determined from the Illumina sequence data and inference from the TA4 phased assembly described above.

### Repeat masking, annotation and quality control

A mango-specific repeat library was created using the strategy described in Repeat Library Construction-Basic in the Maker wiki (http://weatherby.genetics.utah.edu/MAKER/wiki/index.php/Repeat_Library_Construction—Basic). RepeatModeler 1.0.8 (http://www.repeatmasker.org/RepeatModeler.html) and prerequisites RepeatMasker and libraries (http://www.repeatmasker.org), RECON (http://www.reatmasker.org/RECON-1.08.tar.gz), RepeatScout (http://repeatscout.bioprojects.org) TRF (http://tandem.bu.edu/trf/trf.html) and NSEG (ftp://ftp.ncbi.nih.gov/pub/seg/nseg/) were downloaded from the appropriate websites. The mango-specific repeat library and the RepeatMasker libraries cited above were used with RepeatMasker to mask repeats on the TA assembly. Hand curation of potential genes that may have been incorrectly masked revealed only retroviral genes and were subsequently masked prior to annotation.

Annotation of the repeat masked TA4 assembly and overall quality assessment of the annotation was by MAKER-P [66] following the protocol described.

Transcript evidence was from RNA sequence data used in the development of SNP markers [29] as well as five transcriptomes available at NCBI GenBank (GAPC01, GBCV01, GBJO01, GBVW01, GBVX01). Putative protein-coding genes were annotated using the translation data from the transcript evidence (mango_All_Mains_Protein.fasta.txt), uniprot_sprot_plants.fa, TAIR10_pep_20101214_updated, and protein.fa (*Citrus sinensis*). Gene finders were Augustus trained on mango and Evidence Modeler. BUSCO genes were analyzed in the assembly as previously described [67].

### Association of traits to haplotype and genotype of the TaxKP hybrids

Mean fruit weight data for parents and hybrid progeny of the ‘Tommy Atkins’ x ‘Kensington Pride’ population were collected as previously described [13]. The genotype and haplotype at each SNP of each hybrid individual was identified from the phased recombination genetic map. Association of traits to the genotype and haplotype of each hybrid individual at each SNP was accomplished using PLINK [68]. PLINK analysis using qassoc or qassoc.fisher were identical. Initial output was validated with qassoc.perm, which permutated the genotype data for individuals and recalculated the *p*-value of association. The permutated *p*-value estimates were filtered to produce a subset of SNP markers associated with fruit weight at a *p*-value of <= 0.001 that defined the QTL regions for fruit weight.

### Orthofinder analysis

Proteomes of eight species were used for a comparative study against mango. The nine species included are *Arabidopsis* along with four species of climacteric fruits and four species of non-climacteric fruits, namely apple (*Malus domestica*), peach (*Prunus persica*), tomato (*Lycopersicum esculentum*), mango (*Mangifera indica*), orange (*Citrus sinensis*), strawberry *Fragaria x ananassa*), olive (*Olea europaea*), and grape (*Vitus vinifera*). Transposable element-related genes create noise for gene family expansion and contraction analysis, so those genes were identified using TransposonPSI (http://transposonpsi.sourceforge.net) and filtered out. Genes were functionally annotated in orthogroups with Pfam domain ids using kinfin [69] in order to compensate for the high stringency of Orthofinder when assigning orthogroups. Counts were manually merged for orthogroups with similar function and created a list of gene families based on Pfam domains. A total of 3281 gene families were used as input for CAFÉ v3.1 [70] for estimation of lambda values needed to calculate birth and death rate of genes (Supplementary Tables S9 and S10).

### Identification of genes and determination of haplotypes in QTL regions for fruit weight

The nucleotide positions of SNPs in the QTL regions were used to search the annotation of the mango genome assembly to identify candidate genes. The phased genetic map was used to determine the haplotype of the parent donating the heterozygous allele at each SNP in the region and parent haplotypes were summed over the entire QTL region to determine the contribution of each parent. The most common result was that a single haplotype from each parent was observed for the entire QTL region. Presence of more than one haplotype from either maternal or paternal parent was evidence of a recombination event in the region. Perl scripts for this analysis are available upon request. Association of fruit weight with a haplotype was done by ANOVA and a post hoc Tukey’s multiple comparisons of means test.

### Whole genome duplication analysis

Sequence assembly, CDS and gene annotation GFF files were downloaded from NCBI (*P. vera* – Pisver_v2, GCA_008641045.1 [71]) and Phytozome (*C. sinensis* – v1.1, REF JJOQ01000000). WGD (commit a77f8f4 on Nov 26, 2019) was used to estimate the Ks distribution [72]. Timetree (http://timetree.org/) was used to estimate the divergency age between *C. sinensis* and the Anacardiaceae [42].

### Annotation of repetitive sequences

Transposable elements (TEs) in the four genomes (mango, cashew, pistachio, and citrus) were annotated by combining homology-based and de novo-based approaches. For the de novo approach, we used RepeatModeler (Smit et al., 2015), LTR_FINDER [73], LTRharvest [74], and LTR_retriever [75] to build the de novo TE library. For the homology-based approach, we extracted TEs using the Repbase [76] library of each species. TE libraries from these two approaches were combined. We used RepeatMasker [77] against the developed library to identify individual TEs across the selected genomes.

## Supplementary Information

**Additional file 1: Supplemental Data S1**. snpEFF report 'Tommy Atkins'

**Additional file 2: Supplemental Data S2**. snpEFF report 'Kensington Pride'

**Additional file 3: Supplemental Data S3**. Fruit weight LG4 QTL report. **Supplemental Data S4**. Fruit weight LG7 QTL report. **Supplemental Table 1**. Summary of the *M. indica* genome assembly by chromosome. **Supplemental Table 2**. BUSCO analysis for the consensus diploid genome assembly TA4. **Supplemental Table 3**. Occurrence and distribution of repetitive DNA sequences in the mango genome. **Supplemental Table 4**. Protein domain content. **Supplemental Table 5**. Number of homologs pairs with Ks between 0.1 and 0.6 per chromosome. **Supplemental Table 6**. Repetitive elements comparative analysis. **Supplemental Table 7**. Comparison of the SNP variants and variants rate for ‘Tommy Atkins’ and ‘Kensington Pride’ by pseudomolecule. **Supplemental Table S8**. Concurrence of linkage groups between ‘Tommy Atkins’ and ‘Alphonso’. **Supplementary Figure 1**. Repeat composition for *M. indica, P. vera* and *C. sinensis*

**Additional file 4: Supplemental Table S9**, **S10**. Orthofinder reports

## Data Availability

The complete sequence of mango genome was deposited at NCBI under WIOI00000000 accession number. The raw sequence reads that were used to construct the genome are available at Sequence Read Archive (SRA) repository under PRJNA450143.

## Abbreviations

LG: Linkage group
GH1: Glycosyl hydrolases family 1
*M. indica*: *Mangifera indica*
Mt: Metric tons
MYA: Million years ago
PME: Pectin methylesterase
PMEI: Pectin methylesterase inhibitors
PG: Polygalacturonases
QTL: Quantitative trait loci
SNP: Single nucleotide polymorphism
WGD: Whole genome duplication
