# Supplementary material for: The ‘Tommy Atkins’ mango genome reveals candidate genes for fruit quality
Source: BMC Plant Biol. 2021 Feb 22;21:108. doi: 10.1186/s12870-021-02858-1 (PMC7898432; doi:10.1186/s12870-021-02858-1)
Supplement: Supplementary file 1 — Additional file 1: Supplemental Data S1. snpEFF report 'Tommy Atkins' [file 12870_2021_2858_MOESM1_ESM.pdf]

SnpEff: Variant analysis

Contents

[Summary](#)
[Variant rate by chromosome](#)
[Variants by type](#)
[Number of variants by impact](#)
[Number of variants by functional class](#)
[Number of variants by effect](#)
[Quality histogram](#)
[InDel length histogram](#)
[Base variant table](#)
[Transition vs transversions (ts/tv)](#)
[Allele frequency](#)
[Allele Count](#)
[Codon change table](#)
[Amino acid change table](#)
[Chromosome variants plots](#)
[Details by gene](#)

Summary

**Genome**	TA4
**Date**	2018-01-01 17:21
**SnpEff version**	`SnpEff 4.3p (build 2017-06-06 09:55), by Pablo Cingolani`
**Command line arguments**	`SnpEff  TA4 TA4_selfvariant.vcf`
**Warnings**	128,022
**Errors**	0
**Number of lines (input file)**	5,120,815
**Number of variants (before filter)**	5,394,970
**Number of not variants (i.e. reference equals alternative)**	0
**Number of variants processed (i.e. after filter and non-variants)**	5,394,956
**Number of known variants (i.e. non-empty ID)**	0 (0%)
**Number of multi-allelic VCF entries (i.e. more than two alleles)**	4,368
**Number of effects**	10,095,863
**Genome total length**	377,290,333
**Genome effective length**	377,290,333
**Variant rate**	1 variant every 69 bases

Variants rate details

Chromosome	Length	Variants	Variants rate
1	17,320,008	247,141	70
2	17,063,873	260,967	65
3	21,566,805	288,912	74
4	22,357,487	255,999	87
5	14,540,018	227,490	63
6	10,680,009	134,363	79
7	13,133,232	218,581	60
8	14,750,018	223,076	66
9	21,055,410	317,903	66
10	11,063,414	146,226	75
11	17,675,019	197,115	89
12	14,336,529	227,439	63
13	15,099,493	205,623	73
14	13,335,999	208,487	63
15	16,178,320	219,588	73
16	21,434,198	329,642	65
17	11,746,059	157,361	74
18	16,863,820	231,421	72
19	22,398,858	349,731	64
20	16,105,987	443,146	36
10000001	48,585,777	504,745	96
**Total**	**377,290,333**	**5,394,956**	**69**

Number variants by type

Type	Total
**SNP**	3,946,854
**MNP**	1,051,633
**Total**	**5,394,956**

Type	Total
INS	138,977
DEL	146,654
MIXED	110,838
INV	0
DUP	0
BND	0
INTERVAL	0
Total	5,394,956

Number of effects by impact

Type (alphabetical order)	Count	Percent
HIGH	8,352	0.083%
LOW	136,667	1.354%
MODERATE	149,461	1.48%
MODIFIER	9,801,383	97.083%

Number of effects by functional class

Type (alphabetical order)	Count	Percent
MISSENSE	129,328	53.791%
NONSENSE	1,484	0.617%
SILENT	109,615	45.592%

Missense / Silent ratio: 1.1798

Number of effects by type and region

Type

Type (alphabetical order)	Count	Percent
3_prime_UTR_variant	72,021	0.711%
5_prime_UTR_premature_start_codon_gain_variant	5,080	0.05%
5_prime_UTR_variant	42,434	0.419%
conservative_inframe_deletion	815	0.008%
conservative_inframe_insertion	750	0.007%
disruptive_inframe_deletion	514	0.005%
disruptive_inframe_insertion	374	0.004%
downstream_gene_variant	2,123,362	20.973%
frameshift_variant	3,189	0.031%
initiator_codon_variant	30	0%
intergenic_region	4,125,552	40.749%
intragenic_variant	156	0.002%
intron_variant	1,065,055	10.52%
missense_variant	148,074	1.463%
non_coding_transcript_variant	452	0.004%
splice_acceptor_variant	1,304	0.013%
splice_donor_variant	1,307	0.013%
splice_region_variant	25,421	0.251%
start_lost	336	0.003%
stop_gained	2,021	0.02%
stop_lost	496	0.005%
stop_retained_variant	195	0.002%
synonymous_variant	112,764	1.114%
upstream_gene_variant	2,392,499	23.631%

Region

Type (alphabetical order)	Count	Percent
DOWNSTREAM	2,123,353	21.032%
EXON	265,703	2.632%
INTERGENIC	4,125,552	40.864%
INTRON	1,045,061	10.351%
SPLICE_SITE_ACCEPTOR	1,271	0.013%
SPLICE_SITE_DONOR	1,251	0.012%
SPLICE_SITE_REGION	21,175	0.21%
TRANSCRIPT	608	0.006%
UPSTREAM	2,392,499	23.698%
UTR_3_PRIME	71,932	0.712%
UTR_5_PRIME	47,458	0.47%

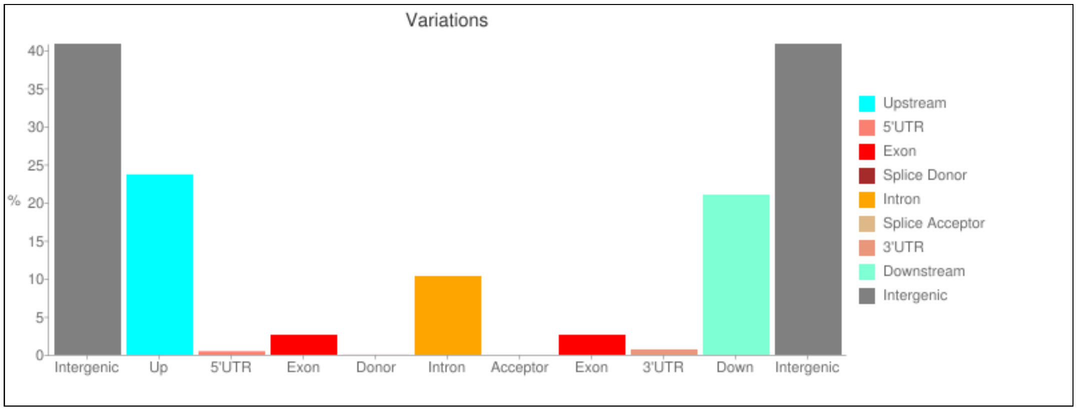

Quality:

**Min**	0
**Max**	2,556,390
**Mean**	5,139.301
**Median**	4,691
**Standard deviation**	25,296.375
**Values**	0,1,2,3,4,5,6,7,8,9,10,11,12,13,14,15,16,17,18,19,20,21,22,23,24,25,26,27,28,29,30,31,32,33,34,35,36,37,38,39,40,41,42,43,44,45,46,47,48,49,50,51,5[illegible]
**Count**	1583,142,98,85,74,50,54,42,61,58,48,63,60,51,42,50,54,55,55,53,61,75,40,58,57,40,55,55,47,46,58,61,60,51,70,57,50,69,55,45,46,46,58,58,44,57,56,[illegible]

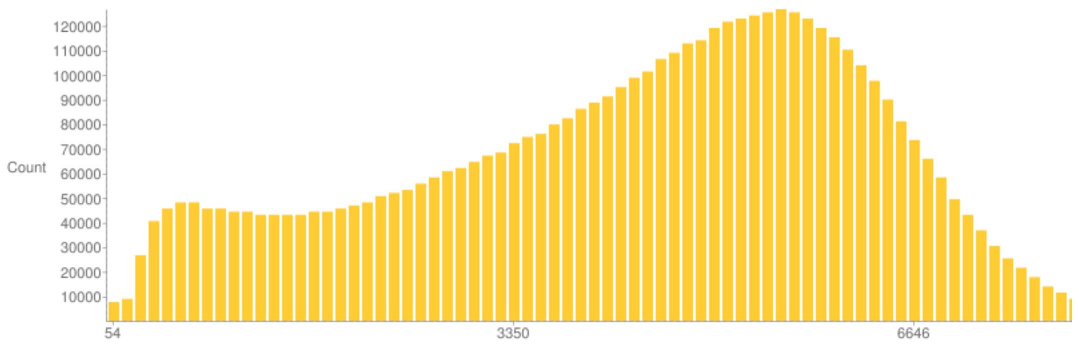

Insertions and deletions length:

**Min**	0
**Max**	37
**Mean**	1.435
**Median**	1
**Standard deviation**	2.626
**Values**	0,1,2,3,4,5,6,7,8,9,10,11,12,13,14,15,16,17,18,19,20,21,22,23,24,25,26,27,28,29,30,32,33,35,37
**Count**	82444,164847,8613,5907,3582,3271,1972,2294,2011,1960,1575,1756,1079,911,744,622,463,422,299,236,190,130,100,67,44,41,23,14,3,1,3,1,1,3,2

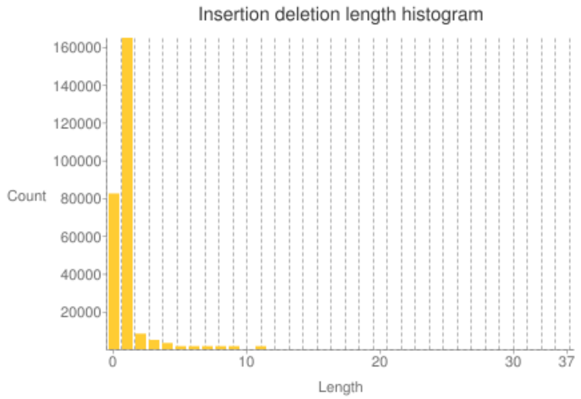

Base changes (SNPs)

	A	C	G	T
**A**	0	142,355	691,920	196,913
**C**	142,764	0	99,547	697,961
**G**	699,883	99,884	0	143,020
**T**	197,267	693,330	142,010	0

Ts/Tv (transitions / transversions)

Note: Only SNPs are used for this statistic.
Note: This Ts/Tv ratio is a 'raw' ratio (ratio of observed events).

**Transitions**	2,768,385
**Transversions**	1,157,453
**Ts/Tv ratio**	2.3918

All variants:

```
Sample ,unknown,Total
Transitions ,2768385,2768385
Transversions ,1157453,1157453
Ts/Tv ,2.392,2.392
```

Only known variants (i.e. the ones having a non-empty ID field):

```
No results available (empty input?)
```

Allele frequency

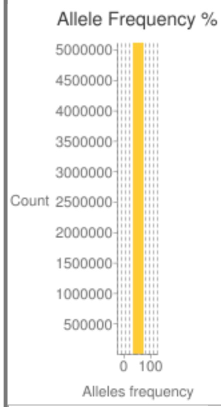

**Min**	0
**Max**	100
**Mean**	50.11
**Median**	50
**Standard deviation**	2.342
**Values**	0,50,100
**Count**	10,5109556,11249

Allele Count

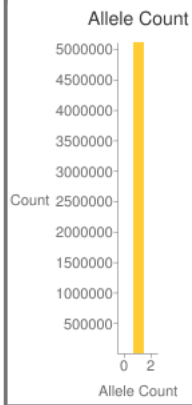

**Min**	0
**Max**	2
**Mean**	1.002
**Median**	1
**Standard deviation**	0.047
**Values**	0,1,2
**Count**	10,5109556,11249

Hom/Het per sample

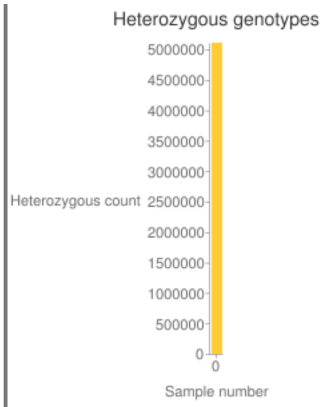

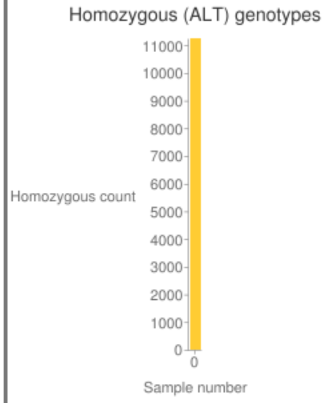

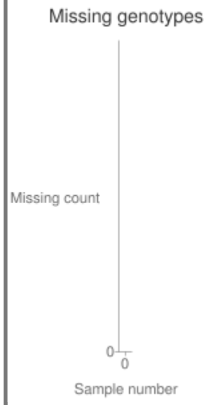


```
Sample_names , unknown
Reference , 10
Het , 5109556
Hom , 11249
Missing , 0
```

Codon changes

How to read this table:
- Rows are reference codons and columns are changed codons. E.g. Row 'AAA' column 'TAA' indicates how many 'AAA' codons have been replaced by 'TAA' codons.
- Red background colors indicate that more changes happened (heat-map).
- Diagonals are indicated using grey background color
- WARNING: This table may include different translation codon tables (e.g. mamalian DNA and mitochondrial DNA).

	-	AAA	AAC	AAG	AAT	ACA	ACC	ACG	ACT	AGA	AGC	AGG	AGT	ATA	ATC	ATG	ATT	CAA	CAC	CAG	CAT
**-**		81	54	89	90	49	31	15	56	45	33	31	64	36	23	55	48	75	45	59	80
**AAA**	233	**117**	373	2,017	518	373	3	4	3	992	4	17	6	213	5	7	11	471	1	8	6
**AAC**	111	403	**60**	399	2,037	6	196	3	4	7	809	6	11	2	120	2	5	13	145	3	6
**AAG**	202	1,985	408	**95**	452	20	2	224	1	21	5	1,014	10	5	3	318	8	6	4	356	2
**AAT**	151	499	2,068	414	**83**	7	7	4	369	9	23	13	1,096	7	7	4	278	5	3	1	354
**ACA**	104	349	8	8	5	**59**	415	1,573	574	293	5	5	4	661	9	62	5	5			1
**ACC**	79	2	224	3	7	396	**35**	271	1,630		265	2	5	4	327	8	15		1		
**ACG**	27	4	6	231	6	1,547	295	**10**	422	1	3	141	2	24	9	825	7			3	
**ACT**	99	7	18	5	386	594	1,723	429	**47**	1	5	1	493	5	12	12	783		2		3
**AGA**	92	1,002	2	25	9	264		3	5	**46**	170	1,032	194	189	4	4	2	13		2	
**AGC**	62	17	760	9	15	6	241	3	11	168	**52**	148	1,236	1	122	2	9		5		
**AGG**	68	22	1	962	5	7		133	2	1,049	164	**30**	184	2	3	149	5			5	1

	-	AAA	AAC	AAG	AAT	ACA	ACC	ACG	ACT	AGA	AGC	AGG	AGT	ATA	ATC	ATG	ATT	CAA	CAC	CAG	CAT
**AGT**	98	6	15	12	1,124	4	5	5	495	177	1,217	202	**51**	1		4	288	1			11
**ATA**	58	195	1	10	8	616	5	16	4	173	2	3	4	**32**	445	879	617	10	1		
**ATC**	58	6	94	1	9	7	336	5	13		93	1	2	487	**29**	269	1,750				1
**ATG**	124	8	1	341	24	58	6	848	15	3	3	201	7	1,004	288	**65**	421			3	1
**ATT**	122	5	7	2	297	4	8	14	772	4	7	3	271	568	1,762	378	**90**				6
**CAA**	119	482	3	12	1	5	1		1	8	2			5				**60**	231	1,586	279
**CAC**	56	2	146		9	1					2					1		184	**22**	249	1,072
**CAG**	92	7	1	340	3	1	2			3	1	10	1	1	2	2		1,528	256	**42**	274
**CAT**	103	8	6	3	340	1			4				10			1	4	316	1,118	263	**49**
**CCA**	135	5	1			157	2		6	2				7				250	3	13	2
**CCC**	77		1			2	90	1	4		1				3			3	56	1	2
**CCG**	61			4		4		53	2			1				1		7	2	142	
**CCT**	142				3	2	4		253	1			4			1	3		4	3	200
**CGA**	35	10				2				356	3	4		1	1	1		1,027	5	14	14
**CGC**	22	1	6							1	101	1	1		4			5	465	5	14
**CGG**	19			5	1			3		12	1	270	1	1	1			15	3	908	9
**CGT**	34	1			11	2			1	7	5	2	138				4	11	13	5	974
**CTA**	47	1				6				1				187		3	6	193		3	3
**CTC**	70		1				2				1		2	3	166	2	7	3	83	2	3
**CTG**	61	1	1	4	1	1		2				3		2		282	7	3		222	1
**CTT**	112		2	1					13				3	6	4	2	391	5		5	226
**GAA**	253	1,470	5	31	13	12		1		29		1	1	7	1	2	1	517	1	3	3
**GAC**	104	8	621	11	23		8	1	2	1	15	3			3	2			127		4
**GAG**	178	18	13	1,166	14			4	1	9		21	2			8		7	1	529	3
**GAT**	257	7	23	13	1,370		1	1	3	4	3		13			1	12	1	5	5	365
**GCA**	118	13				1,160	10	15	8	10	3		1	22				11	1		
**GCC**	96		14	2		4	643	2	18	1	8				11		2		1	1	
**GCG**	32			5		15	2	225	5	2		3		1		18				3	
**GCT**	108		1		8	4	29	3	1,242	1	2	1	5	2	1		29	1		1	5
**GGA**	164	28		1	1	1			1	726	3	14	8	5		3	1	9			
**GGC**	88		19	1	2		3			3	580	5	15		3				5		
**GGG**	94	1	3	14			1	3		5	2	444	7			1		1		15	
**GGT**	160	2	3	1	25				8	2	11	3	996		1	2	5		1		13
**GTA**	54	3		1		18	3		1	8	1		1	847	6	12	10	2			1
**GTC**	35		2				8				2		1	5	753	5	14			1	1
**GTG**	75	2		7		4		17				1	2	20	7	882	3		1	2	
**GTT**	129	2			6	3			18	1			13	8	18	5	1,644		1		1
**TAA**	19	24																38		1	
**TAC**	53	5	129	3	6		2	1			3				2			2	334	2	9
**TAG**	13			13																42	
**TAT**	96	2	6		290	2					1		7	3			2	5	7	1	727
**TCA**	131	6			2	270	4	7	4	1				6	1	1	1	6			1
**TCC**	62					1	151		8		5	1			2		1		1		2
**TCG**	35				1	2	1	50	1	1						9	1				
**TCT**	193	2			15	3	6		325		1		9		4		11	1			13
**TGA**	18									14								5			
**TGC**	42		3		1	1	1			2	107	2	7			1			25		
**TGG**	50			1						2	3	135	2			6				39	1
**TGT**	44				2		1		1		3		234				4	4	3	3	36
**TTA**	80	6	1		2	1			2	4				226	4	6	2	3			1
**TTC**	88		3				1			2				2	162	3	12	1	3	1	
**TTG**	120			5	1	3		4	2			1		5		417	4			7	1
**TTT**	171			1	7		1		9	1			3	7	5	5	369		1	1	12

Amino acid changes

How to read this table:
- Rows are reference amino acids and columns are changed amino acids. E.g. Row 'A' column 'E' indicates how many 'A' amino acids have been replaced by 'E' amino acids.
- Red background colors indicate that more changes happened (heat-map).
- Diagonals are indicated using grey background color
- WARNING: This table may include different translation codon tables (e.g. mamalian DNA and mitochondrial DNA).

	*	-	?	A	C	D	E	F	G	H	I	K	L	M	N	P	Q	R	S	T	V
*****	**209**	42	8		9	2	39	1	8			37	47				86	71	41		
**-**	40		1,381	154	49	148	214	126	168	125	107	170	225	55	144	210	134	122	353	151	[illegible]
**?**																					
**A**	4	339	15	**12,203**	14	424	594	16	1,075	7	68	20	49	18	23	826	17	24	1,330	3,385	[illegible]
**C**	55	76	10	5	**2,001**	7	1	313	252	64	4		31	1	6	8	7	917	830	4	

	*	-	?	A	C	D	E	F	G	H	I	K	L	M	N	P	Q	R	S	T	V
**D**	6	343	18	355	17	**5,148**	2,174	6	1,291	501	15	39	6	3	2,037	4	6	20	48	16	2[illegible]
**E**	185	412	19	547		2,131	**4,238**	4	1,402	8	9	2,685	17	10	45	6	1,056	72	12	18	3[illegible]
**F**	3	243	16	19	330	15	2	**3,982**	4	16	557	1	2,420	8	10	24	3	11	1,019	11	5[illegible]
**G**	67	486	20	1,130	306	1,320	1,398	12	**9,215**	19	15	48	21	6	53	11	25	1,736	1,635	17	7[illegible]
**H**	6	149	10	7	93	439	11	16	24	**2,261**	4	13	326	2	501	239	1,012	1,355	25	6	
**I**	2	219	19	48	1	12	8	514	11	8	**5,780**	219	1,018	1,526	416	11	10	194	391	1,800	3,3[illegible]
**K**	127	407	28	28	1	28	2,429	1	43	13	245	**4,214**	12	325	1,751	2	841	2,061	36	630	[illegible]
**L**	147	445	45	40	22	5	22	2,446	18	318	1,020	18	**18,108**	712	9	1,814	446	471	1,669	36	1,9[illegible]
**M**	2	115	9	12	1	5	10	12	6	1	1,713	349	712	**65**	25	1	3	206	16	927	9[illegible]
**N**	5	244	18	16	14	2,061	47	14	42	508	419	1,715	6	6	**4,248**	5	22	48	1,955	596	[illegible]
**P**	9	390	25	822	6	3	7	17	6	269	13	9	1,857	2	5	**10,285**	419	528	1,939	580	[illegible]
**Q**	405	195	16	13	2	13	1,007		27	1,040	8	841	420	2	8	427	**3,216**	1,931	21	10	
**R**	297	246	24	23	1,036	24	40	10	1,689	1,498	217	2,028	512	154	35	495	2,010	**6,533**	979	422	
**S**	181	547	34	1,347	935	46	12	1,089	1,699	33	448	52	1,770	16	1,932	1,932	8	1,007	**13,924**	1,603	[illegible]
**T**	2	293	16	3,134	5	16	26	14	32	7	1,861	609	35	907	660	623	8	449	1,606	**10,020**	[illegible]
**V**	3	269	24	3,102	9	279	366	662	707	5	3,335	15	1,971	904	8	19	5	19	42	72	**8,8[illegible]**
**W**	269	48	2		207	1	3	8	98	1		1	198	6		2	39	505	110		
**Y**	192	130	19	1	859	411	11	653	7	1,077	7	10	38		431	11	10	24	412	5	

Variants by chromosome

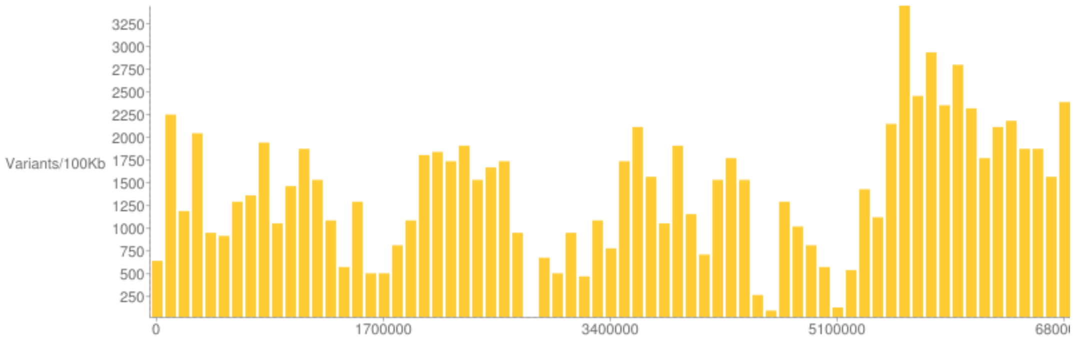

1, Position,0,100000,200000,300000,400000,500000,600000,700000,800000,900000,1000000,1100000,1200000,1300000,1400000,1500000,1600000
1,Count,667,2268,1211,2046,951,920,1290,1374,1943,1054,1474,1898,1538,1110,576,1288,524,506,823,1102,1825,1860,1759,1914,1542,1676,1737,973,20,700,5

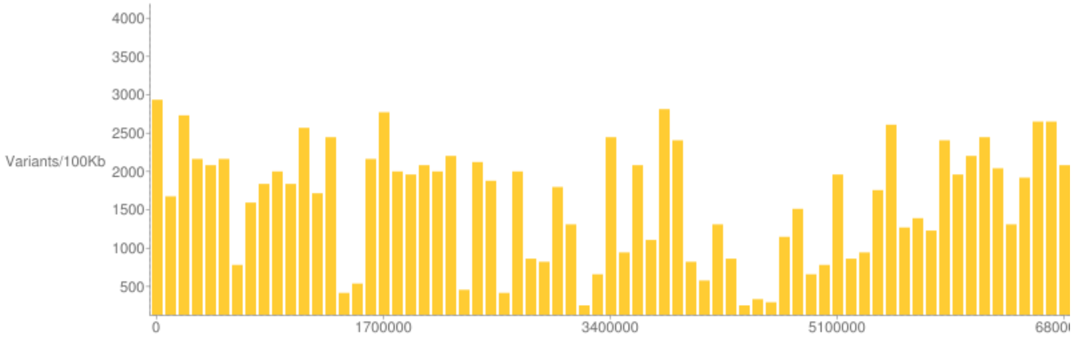

2, Position,0,100000,200000,300000,400000,500000,600000,700000,800000,900000,1000000,1100000,1200000,1300000,1400000,1500000,1600000
2,Count,2958,1695,2761,2161,2099,2194,775,1612,1831,2003,1870,2581,1747,2462,436,567,2168,2770,2024,1978,2083,2002,2198,487,2117,1871,415,1996,868,8

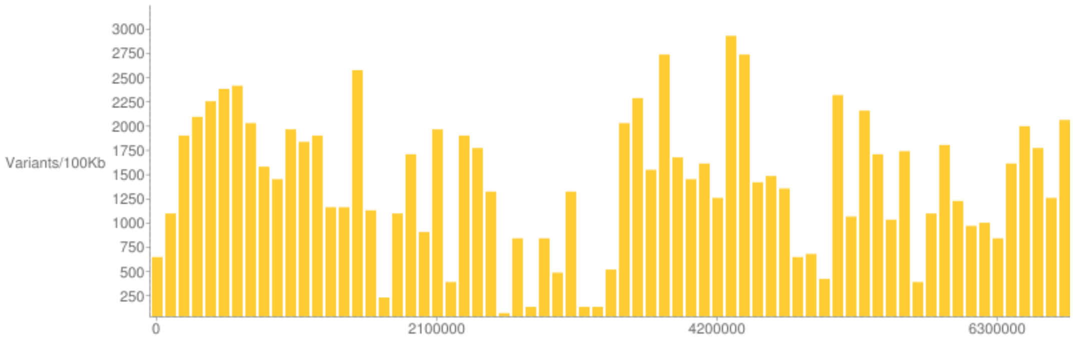


```
3, Position,0,100000,200000,300000,400000,500000,600000,700000,800000,900000,1000000,1100000,1200000,1300000,1400000,1500000,1600000
3,Count,652,1121,1919,2096,2273,2396,2425,2053,1578,1469,1963,1861,1906,1165,1189,2585,1148,240,1125,1704,925,1986,396,1926,1791,1326,72,866,141,867
```

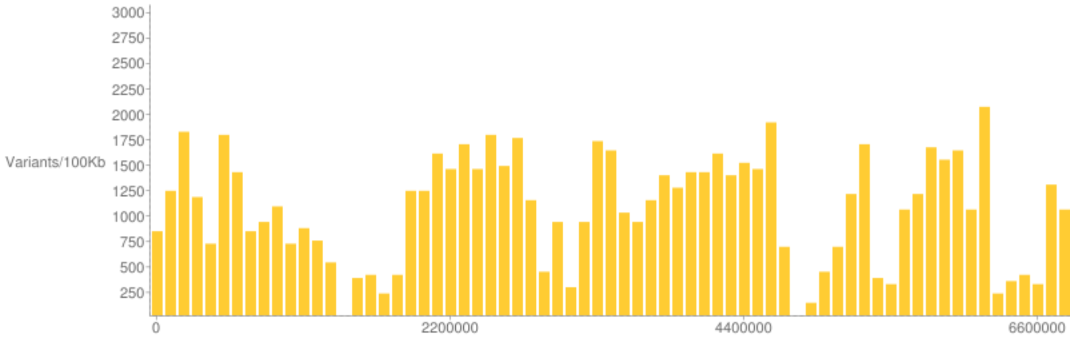


```
4, Position,0,100000,200000,300000,400000,500000,600000,700000,800000,900000,1000000,1100000,1200000,1300000,1400000,1500000,1600000
4,Count,858,1270,1834,1202,745,1794,1438,874,961,1115,747,896,781,560,45,406,434,236,437,1243,1247,1630,1481,1704,1487,1799,1498,1778,1175,476,950,3
```

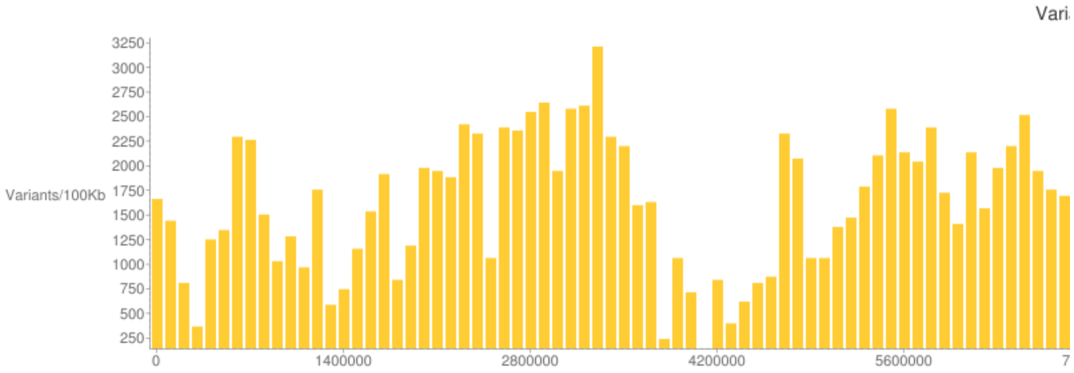


```
5, Position,0,100000,200000,300000,400000,500000,600000,700000,800000,900000,1000000,1100000,1200000,1300000,1400000,1500000,1600000
5,Count,1663,1464,832,377,1255,1350,2294,2267,1520,1041,1300,972,1782,599,767,1171,1536,1938,861,1210,1989,1949,1890,2431,2348,1068,2407,2375,2569,2
```

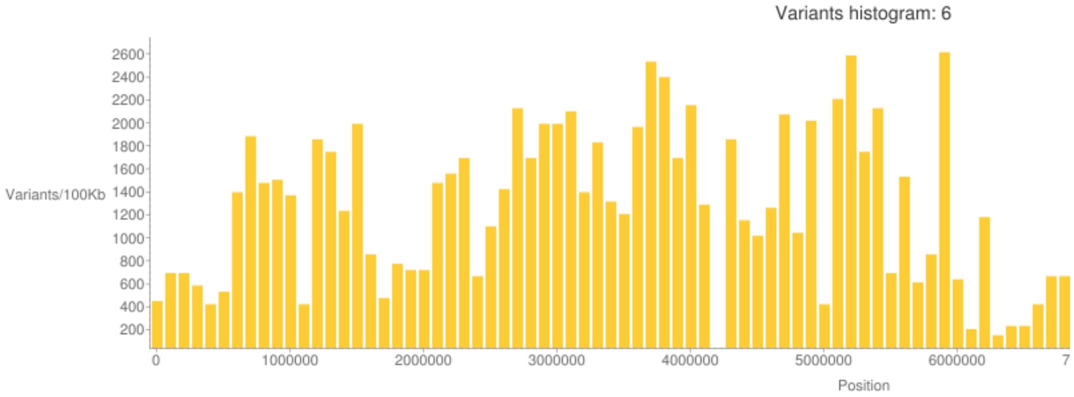


```
6, Position,0,100000,200000,300000,400000,500000,600000,700000,800000,900000,1000000,1100000,1200000,1300000,1400000,1500000,1600000
6,Count,445,714,709,583,439,537,1397,1899,1492,1511,1379,428,1869,1766,1246,2005,850,485,771,735,732,1489,1577,1705,676,1106,1442,2139,1715,2008,198
```

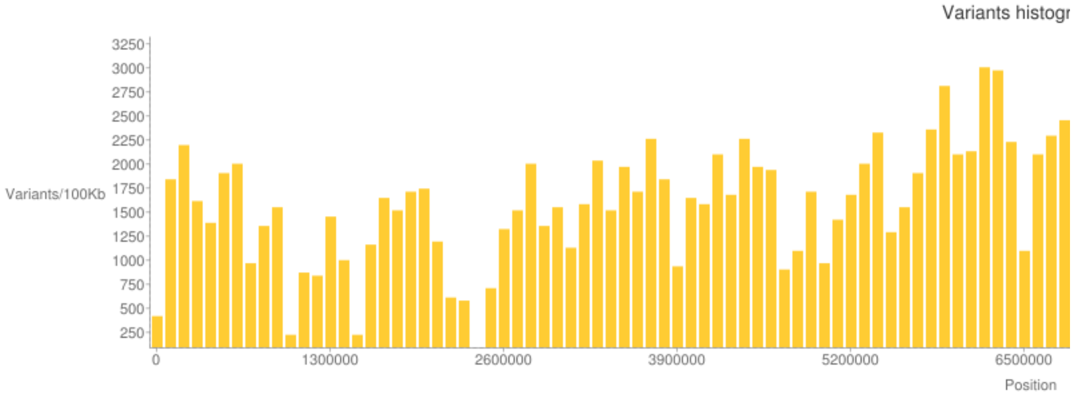


```
7, Position,0,100000,200000,300000,400000,500000,600000,700000,800000,900000,1000000,1100000,1200000,1300000,1400000,1500000,1600000
7,Count,424,1865,2210,1630,1408,1912,2002,975,1380,1574,221,883,857,1451,1005,247,1183,1647,1532,1726,1748,1196,627,592,90,709,1336,1541,2020,1356,1
```

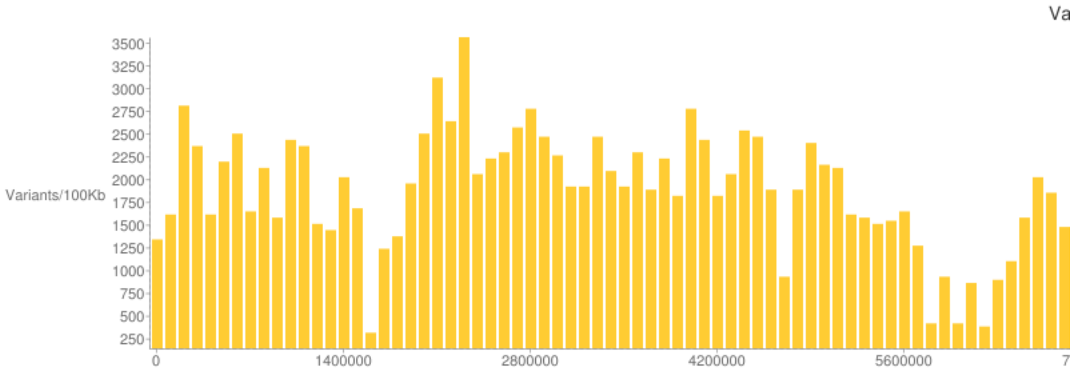


```
8, Position,0,100000,200000,300000,400000,500000,600000,700000,800000,900000,1000000,1100000,1200000,1300000,1400000,1500000,1600000
8,Count,1367,1619,2841,2381,1625,2195,2533,1678,2133,1600,2459,2367,1514,1447,2024,1706,314,1265,1405,1961,2515,3145,2647,3561,2074,2245,2320,2588,2
```

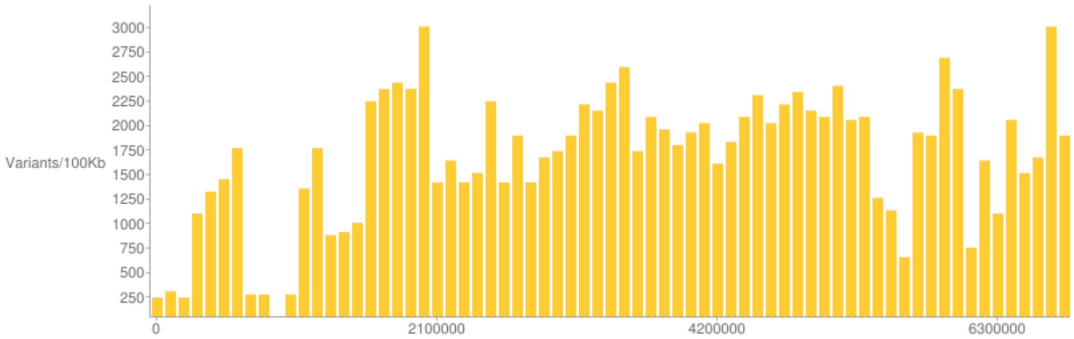

9, Position,0,100000,200000,300000,400000,500000,600000,700000,800000,900000,1000000,1100000,1200000,1300000,1400000,1500000,1600000
9,Count,244,317,256,1111,1322,1476,1788,302,295,77,283,1378,1775,899,934,1030,2243,2371,2435,2385,3022,1436,1663,1438,1533,2267,1420,1914,1424,1686,

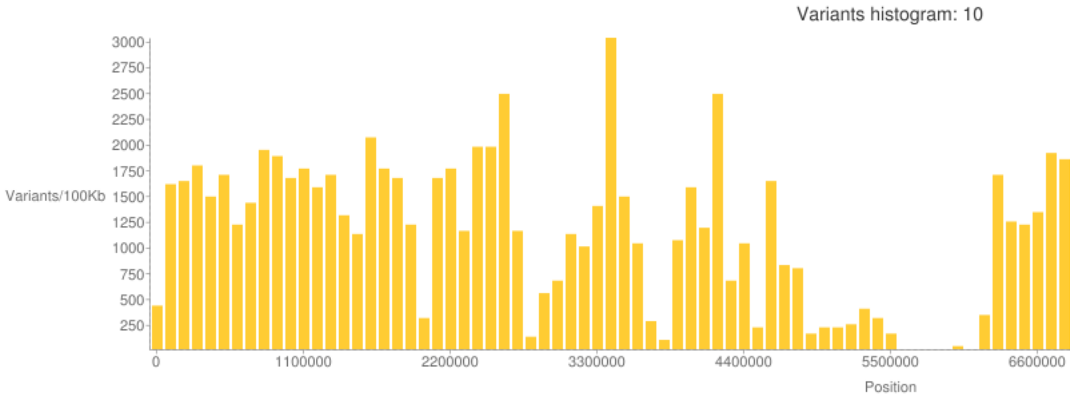

10, Position,0,100000,200000,300000,400000,500000,600000,700000,800000,900000,1000000,1100000,1200000,1300000,1400000,1500000,160000
10,Count,440,1630,1673,1805,1499,1718,1237,1447,1962,1916,1691,1768,1586,1707,1329,1155,2089,1790,1698,1224,324,1682,1793,1175,1980,1989,2521,1180,1

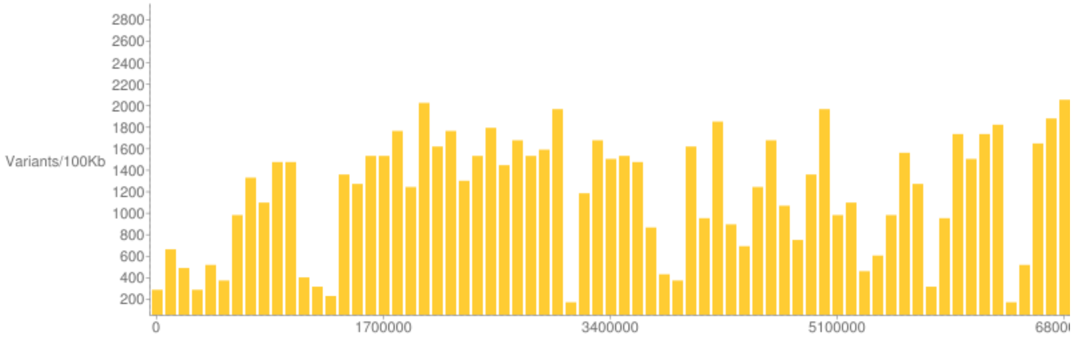

11, Position,0,100000,200000,300000,400000,500000,600000,700000,800000,900000,1000000,1100000,1200000,1300000,1400000,1500000,160000
11,Count,302,672,496,307,514,383,989,1337,1093,1475,1486,422,320,238,1370,1294,1551,1548,1766,1243,2044,1621,1778,1296,1546,1814,1463,1689,1546,1586

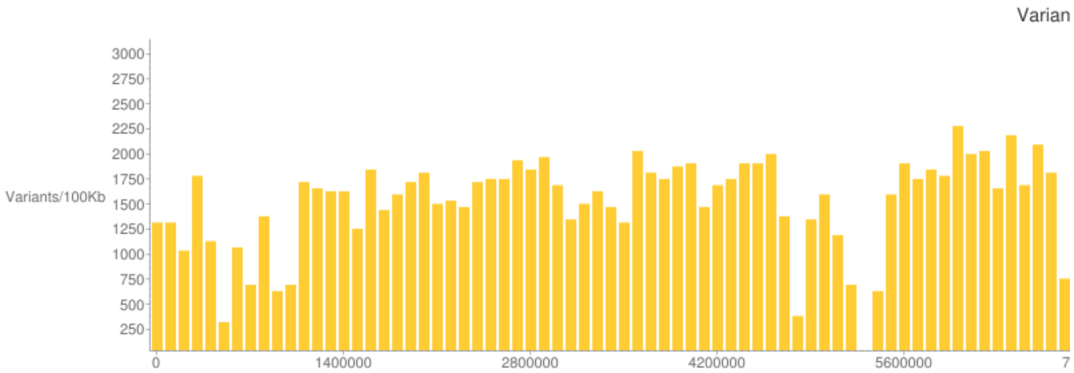

12, Position,0,100000,200000,300000,400000,500000,600000,700000,800000,900000,1000000,1100000,1200000,1300000,1400000,1500000,160000
12,Count,1330,1309,1034,1780,1124,334,1091,689,1388,641,707,1738,1653,1646,1626,1272,1855,1461,1616,1726,1810,1499,1532,1467,1740,1761,1764,1936,185

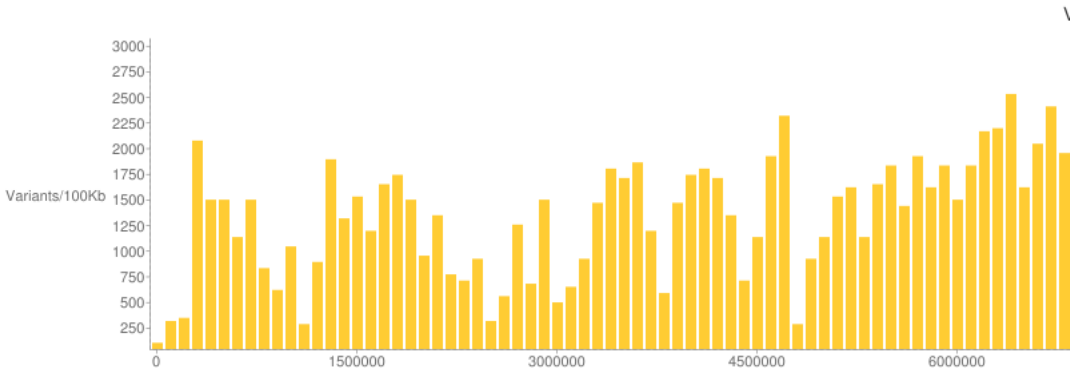

13, Position,0,100000,200000,300000,400000,500000,600000,700000,800000,900000,1000000,1100000,1200000,1300000,1400000,1500000,160000
13,Count,122,324,361,2103,1499,1520,1138,1507,853,622,1060,296,907,1917,1333,1550,1196,1660,1765,1514,952,1373,787,734,938,319,571,1281,678,1506,524

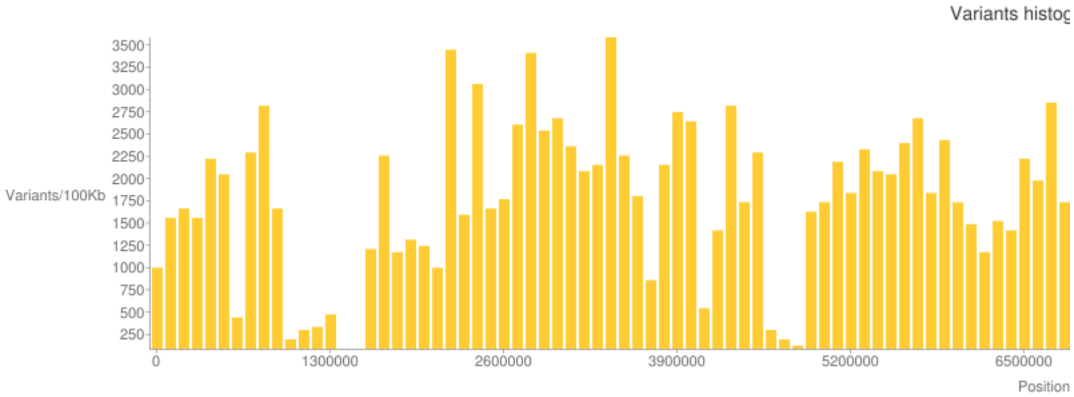

14, Position,0,100000,200000,300000,400000,500000,600000,700000,800000,900000,1000000,1100000,1200000,1300000,1400000,1500000,160000
14,Count,1015,1580,1667,1551,2217,2051,459,2308,2837,1658,193,295,326,472,106,115,1210,2258,1176,1322,1258,1016,3459,1603,3090,1686,1789,2634,3435,2

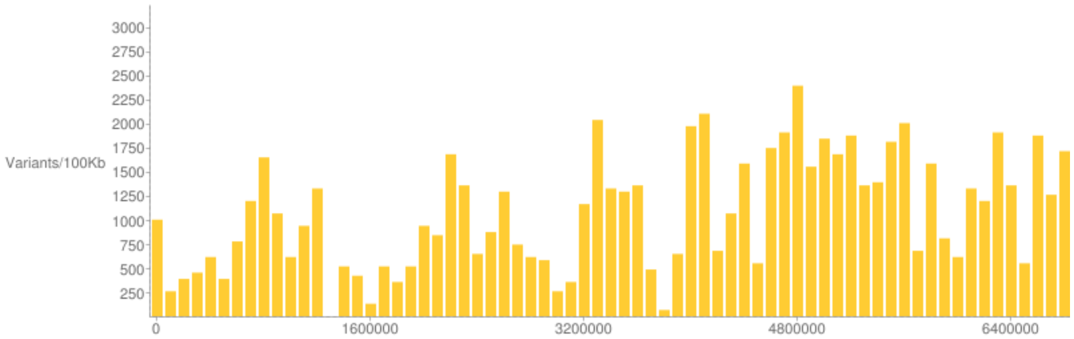


```
15, Position,0,100000,200000,300000,400000,500000,600000,700000,800000,900000,1000000,1100000,1200000,1300000,1400000,1500000,160000
15,Count,1027,293,392,471,644,419,783,1201,1657,1093,643,942,1345,3,544,428,138,543,387,551,960,868,1686,1381,650,900,1304,774,627,614,281,374,1192,
```

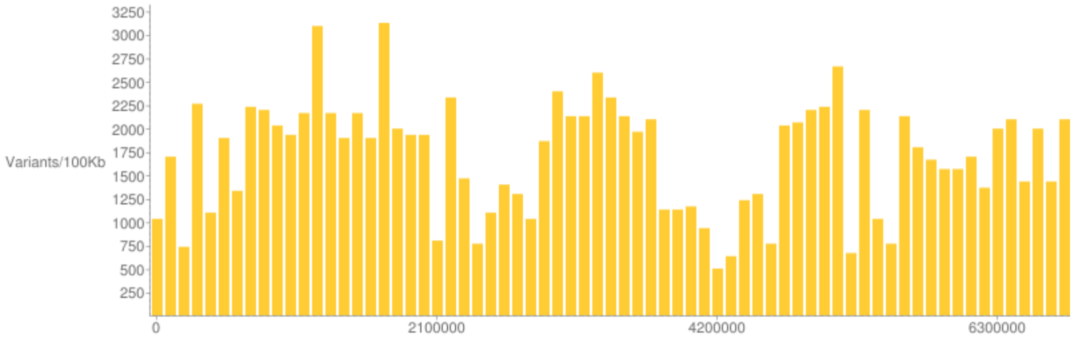


```
16, Position,0,100000,200000,300000,400000,500000,600000,700000,800000,900000,1000000,1100000,1200000,1300000,1400000,1500000,160000
16,Count,1056,1702,769,2274,1117,1904,1357,2242,2204,2035,1964,2175,3114,2194,1901,2195,1918,3156,2018,1959,1937,837,2353,1498,783,1116,1434,1306,10
```

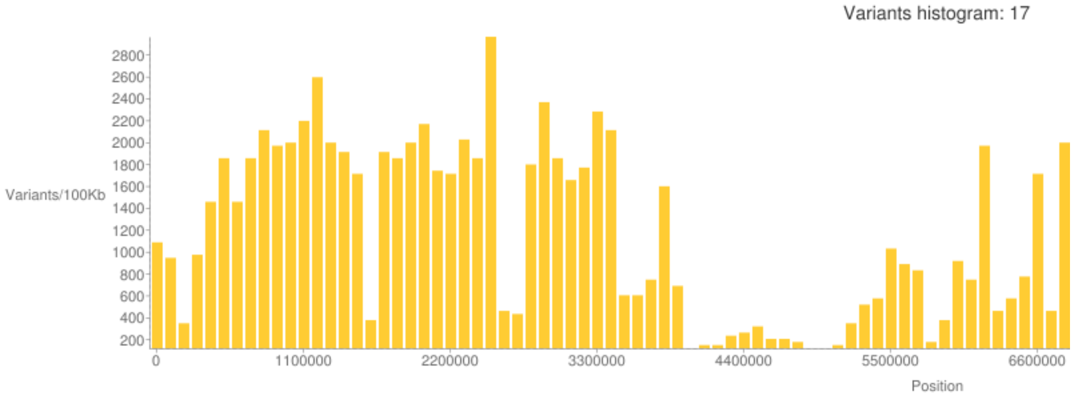


```
17, Position,0,100000,200000,300000,400000,500000,600000,700000,800000,900000,1000000,1100000,1200000,1300000,1400000,1500000,160000
17,Count,1088,963,347,982,1467,1866,1464,1856,2118,1971,2002,2215,2595,2009,1929,1731,388,1937,1872,2018,2174,1753,1736,2041,1864,2962,481,431,1797,
```

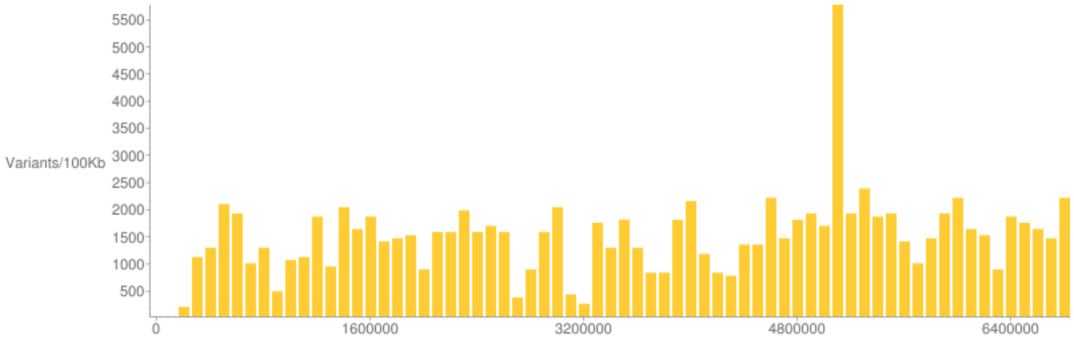


```
18, Position,0,100000,200000,300000,400000,500000,600000,700000,800000,900000,1000000,1100000,1200000,1300000,1400000,1500000,160000
18,Count,26,32,210,1157,1310,2096,1971,1058,1329,525,1082,1118,1896,1000,2038,1666,1885,1405,1495,1522,906,1631,1616,1994,1587,1724,1605,409,938,161
```

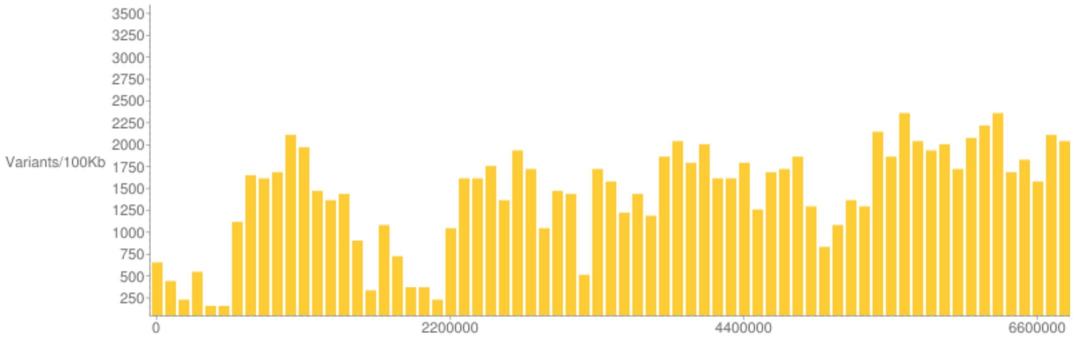


```
19, Position,0,100000,200000,300000,400000,500000,600000,700000,800000,900000,1000000,1100000,1200000,1300000,1400000,1500000,160000
19,Count,659,453,227,575,184,181,1145,1656,1635,1684,2119,1964,1500,1361,1463,921,341,1085,736,383,390,255,1063,1620,1628,1783,1390,1949,1722,1069,1
```

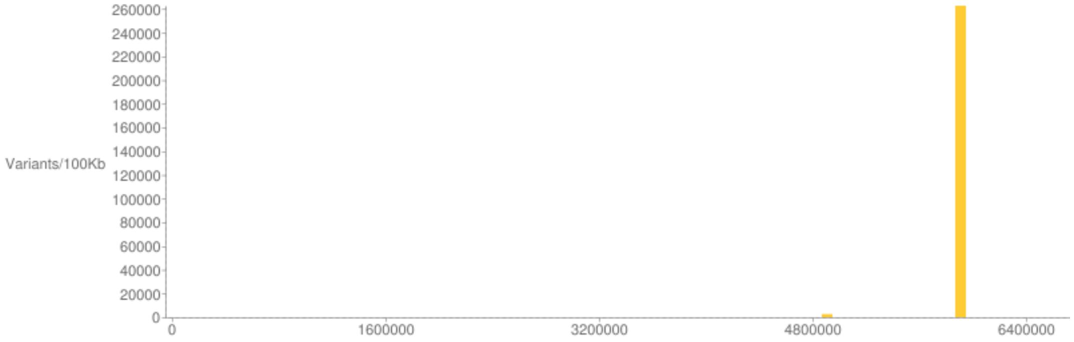


```
20, Position,0,100000,200000,300000,400000,500000,600000,700000,800000,900000,1000000,1100000,1200000,1300000,1400000,1500000,160000
20,Count,13,1420,108,666,1301,357,888,1702,397,1978,1629,869,626,2212,1802,970,223,391,1942,1594,1521,1699,1481,1781,1354,860,6,57,145,711,2332,2332
```

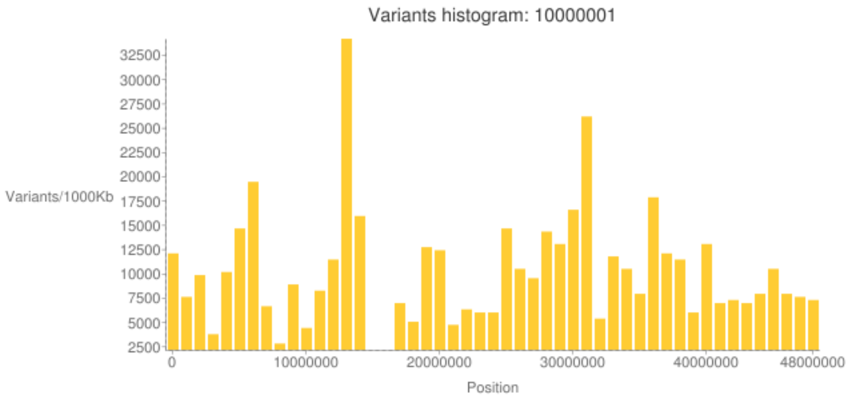


```
10000001, Position,0,1000000,2000000,3000000,4000000,5000000,6000000,7000000,8000000,9000000,10000000,11000000,12000000,13000000,140
10000001,Count,12374,7753,9922,4001,10389,14734,19488,6680,2819,9073,4547,8303,11689,34154,16178,2131,2350,6973,5200,12938,12571,4773,6473,6113,6212
```

Details by gene

[Here]() you can find a tab-separated table.
