# Supplementary material for: The ‘Tommy Atkins’ mango genome reveals candidate genes for fruit quality
Source: BMC Plant Biol. 2021 Feb 22;21:108. doi: 10.1186/s12870-021-02858-1 (PMC7898432; doi:10.1186/s12870-021-02858-1)
Supplement: Supplementary file 2 — Additional file 2: Supplemental Data S2. snpEFF report 'Kensington Pride' [file 12870_2021_2858_MOESM2_ESM.pdf]

SnpEff: Variant analysis

Contents

Summary
Variant rate by chromosome
Variants by type
Number of variants by impact
Number of variants by functional class
Number of variants by effect
Quality histogram
InDel length histogram
Base variant table
Transition vs transversions (ts/tv)
Allele frequency
Allele Count
Codon change table
Amino acid change table
Chromosome variants plots
Details by gene

Summary

**Genome**	TA4
**Date**	2018-01-01 19:47
**SnpEff version**	`SnpEff 4.3p (build 2017-06-06 09:55), by Pablo Cingolani`
**Command line arguments**	`SnpEff  TA4 kp_all.vcf`
**Warnings**	201,439
**Errors**	0
**Number of lines (input file)**	7,791,116
**Number of variants (before filter)**	9,030,162
**Number of not variants (i.e. reference equals alternative)**	0
**Number of variants processed (i.e. after filter and non-variants)**	9,030,142
**Number of known variants (i.e. non-empty ID)**	0 (0%)
**Number of multi-allelic VCF entries (i.e. more than two alleles)**	450,364
**Number of effects**	17,539,335
**Genome total length**	377,290,333
**Genome effective length**	377,290,333
**Variant rate**	1 variant every 41 bases

Variants rate details

Chromosome	Length	Variants	Variants rate
1	17,320,008	466,510	37
2	17,063,873	428,844	39
3	21,566,805	530,741	40
4	22,357,487	484,241	46
5	14,540,018	324,800	44
6	10,680,009	206,656	51
7	13,133,232	361,416	36
8	14,750,018	356,138	41
9	21,055,410	481,402	43
10	11,063,414	222,749	49
11	17,675,019	690,820	25
12	14,336,529	310,197	46
13	15,099,493	357,215	42
14	13,335,999	328,449	40
15	16,178,320	329,319	49
16	21,434,198	430,451	49
17	11,746,059	324,153	36
18	16,863,820	401,989	41
19	22,398,858	800,148	27
20	16,105,987	621,593	25
10000001	48,585,777	572,311	84
**Total**	**377,290,333**	**9,030,142**	**41**

Number variants by type

Type	Total
**SNP**	6,291,666
**MNP**	1,568,959
**Total**	**9,030,142**

Type	Total
INS	223,249
DEL	245,632
MIXED	700,636
INV	0
DUP	0
BND	0
INTERVAL	0
Total	9,030,142

Number of effects by impact

Type (alphabetical order)	Count	Percent
HIGH	13,392	0.076%
LOW	203,108	1.158%
MODERATE	235,851	1.345%
MODIFIER	17,086,984	97.421%

Number of effects by functional class

Type (alphabetical order)	Count	Percent
MISSENSE	201,746	55.063%
NONSENSE	2,688	0.734%
SILENT	161,956	44.203%

Missense / Silent ratio: 1.2457

Number of effects by type and region

Type

Type (alphabetical order)	Count	Percent
3_prime_UTR_variant	110,313	0.627%
5_prime_UTR_premature_start_codon_gain_variant	7,617	0.043%
5_prime_UTR_truncation	1	0%
5_prime_UTR_variant	65,666	0.373%
bidirectional_gene_fusion	2	0%
conservative_inframe_deletion	1,268	0.007%
conservative_inframe_insertion	1,127	0.006%
disruptive_inframe_deletion	878	0.005%
disruptive_inframe_insertion	560	0.003%
downstream_gene_variant	4,136,549	23.526%
exon_loss_variant	3	0%
frameshift_variant	5,015	0.029%
initiator_codon_variant	73	0%
intergenic_region	7,001,925	39.822%
intragenic_variant	284	0.002%
intron_variant	1,725,408	9.813%
missense_variant	233,638	1.329%
non_coding_transcript_variant	775	0.004%
splice_acceptor_variant	2,024	0.012%
splice_donor_variant	2,005	0.011%
splice_region_variant	39,208	0.223%
start_lost	572	0.003%
stop_gained	3,519	0.02%
stop_lost	758	0.004%
stop_retained_variant	317	0.002%
synonymous_variant	166,397	0.946%
upstream_gene_variant	4,076,994	23.187%

Region

Type (alphabetical order)	Count	Percent
DOWNSTREAM	4,136,532	23.584%
EXON	408,289	2.328%
GENE	2	0%
INTERGENIC	7,001,925	39.921%
INTRON	1,694,663	9.662%
SPLICE_SITE_ACCEPTOR	1,965	0.011%
SPLICE_SITE_DONOR	1,920	0.011%
SPLICE_SITE_REGION	32,557	0.186%
TRANSCRIPT	1,059	0.006%
UPSTREAM	4,076,994	23.245%
UTR_3_PRIME	110,222	0.628%
UTR_5_PRIME	73,207	0.417%

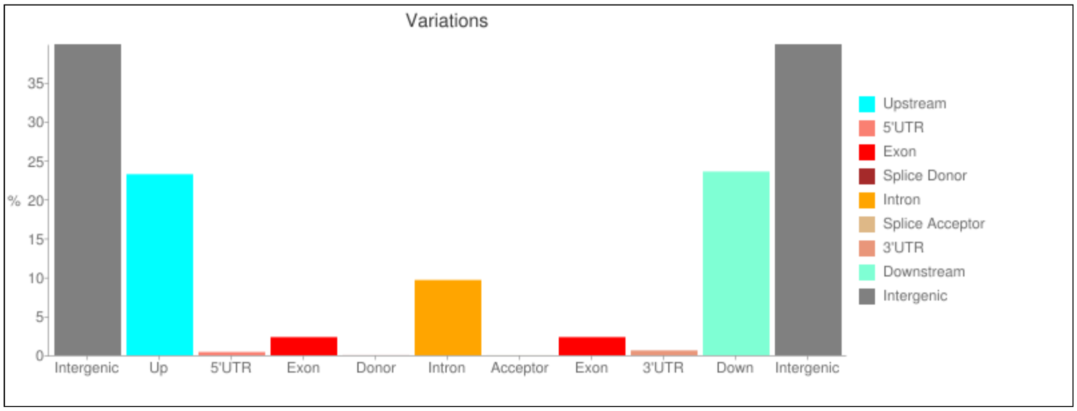

Quality:

**Min**	0
**Max**	6,264,490
**Mean**	5,818.445
**Median**	4,225
**Standard deviation**	49,780.119
**Values**	0,1,2,3,4,5,6,7,8,9,10,11,12,13,14,15,16,17,18,19,20,21,22,23,24,25,26,27,28,29,30,31,32,33,34,35,36,37,38,39,40,41,42,43,44,45,46,47,48,49,50,51,5
**Count**	25489,3136,2077,1860,1498,1297,1280,1274,1204,1139,1063,1191,1076,1151,1025,1046,1109,1053,1093,1065,1127,1096,1091,1073,1092,1182,1071

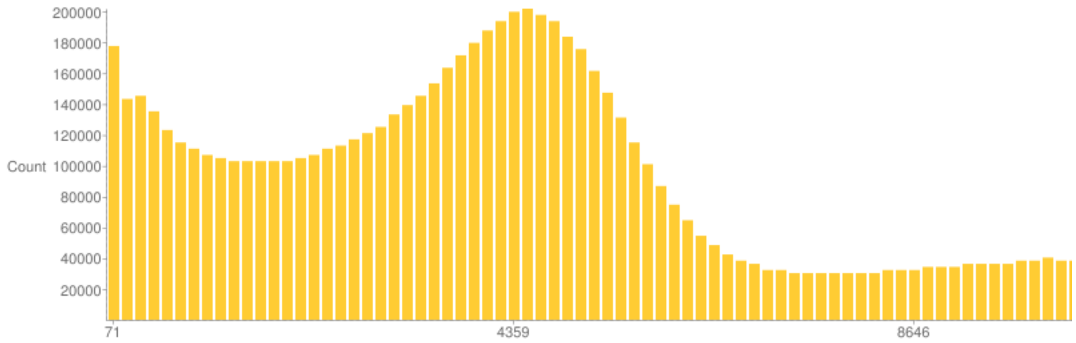

Insertions and deletions length:

**Min**	0
**Max**	34
**Mean**	1.467
**Median**	1
**Standard deviation**	2.71
**Values**	0,1,2,3,4,5,6,7,8,9,10,11,12,13,14,15,16,17,18,19,20,21,22,23,24,25,26,27,28,29,30,31,32,34
**Count**	129104,275837,14654,10044,5974,5428,3173,3676,3183,3229,2504,2784,1787,1435,1207,993,710,698,508,457,387,267,218,166,132,102,76,57,30,33

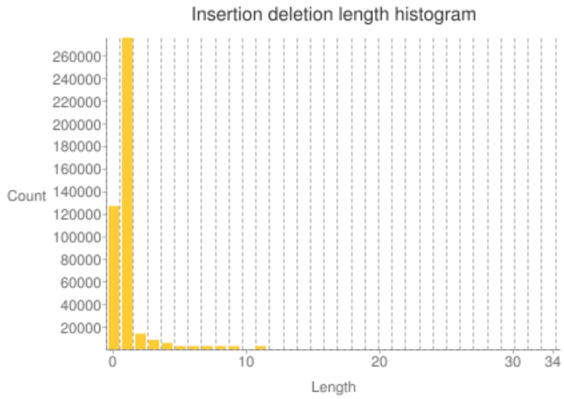

Base changes (SNPs)

	A	C	G	T

**A**	0	222,604	1,077,667	312,402
**C**	227,322	0	152,618	1,151,534
**G**	1,151,752	152,831	0	227,746
**T**	311,943	1,080,437	222,810	0

Ts/Tv (transitions / transversions)

Note: Only SNPs are used for this statistic.
Note: This Ts/Tv ratio is a 'raw' ratio (ratio of observed events).

**Transitions**	5,388,710
**Transversions**	2,200,036
**Ts/Tv ratio**	2.4494

All variants:

```
Sample ,unknown,Total
Transitions ,5388710,5388710
Transversions ,2200036,2200036
Ts/Tv ,2.449,2.449
```

Only known variants (i.e. the ones having a non-empty ID field):

```
No results available (empty input?)
```

Allele frequency

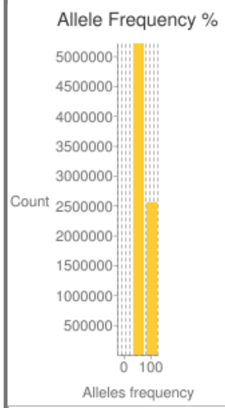

**Min**	0
**Max**	100
**Mean**	66.544
**Median**	50
**Standard deviation**	23.527
**Values**	0,50,100
**Count**	17,5213172,2577927

Allele Count

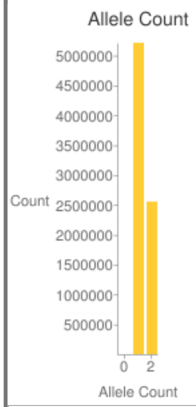

**Min**	0
**Max**	2
**Mean**	1.331
**Median**	1
**Standard deviation**	0.471
**Values**	0,1,2
**Count**	17,5213172,2577927

Hom/Het per sample

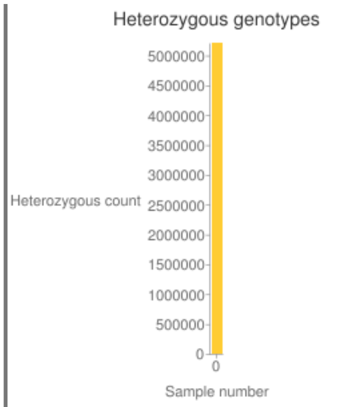

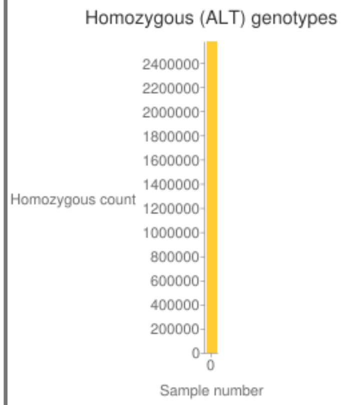

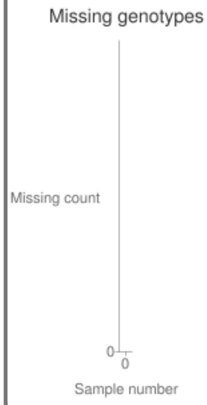


```
Sample_names , unknown
Reference , 17
Het , 5213172
Hom , 2577927
Missing , 0
```

Codon changes

How to read this table:
- Rows are reference codons and columns are changed codons. E.g. Row 'AAA' column 'TAA' indicates how many 'AAA' codons have been replaced by 'TAA' codons.
- Red background colors indicate that more changes happened (heat-map).
- Diagonals are indicated using grey background color
- WARNING: This table may include different translation codon tables (e.g. mamalian DNA and mitochondrial DNA).

	-	AAA	AAC	AAG	AAT	ACA	ACC	ACG	ACT	AGA	AGC	AGG	AGT	ATA	ATC	ATG	ATT	CAA	CAC	CAG	CAT
**-**		119	73	121	129	75	55	24	92	69	52	62	83	41	37	67	71	118	66	111	[illegible]
**AAA**	357	**184**	575	3,011	796	559	6	11	6	1,580	11	29	17	355	5	18	13	797	5	15	[illegible]
**AAC**	193	655	**96**	618	3,037	7	312	3	17	9	1,241	7	30	4	184	3	3	15	212	7	[illegible]
**AAG**	311	3,088	660	**140**	787	23	6	382	11	29	17	1,577	4	8	7	545	8	18	6	548	[illegible]
**AAT**	271	820	3,046	640	**155**	10	9	6	579	12	39	16	1,764	6	16	8	488	14	7	4	[illegible]
**ACA**	151	562	12	14	11	**88**	593	2,351	915	472	7	7	9	1,102	12	115	10	10			[illegible]
**ACC**	106	2	391	6	4	530	**41**	440	2,363	1	411	3	12	11	529	10	24		2		
**ACG**	45	11	5	368	7	2,329	422	**17**	579	7	2	201	4	32	6	1,298	14			3	[illegible]
**ACT**	155	14	19	5	601	987	2,610	642	**87**	6	10	4	777	11	19	23	1,307		3	3	[illegible]
**AGA**	170	1,540	4	44	18	432		10	5	**100**	265	1,506	262	317	3	5	5	20	1	2	
**AGC**	86	21	1,168	16	27	8	380	4	16	254	**84**	218	1,882	4	180	1	12		6	1	[illegible]
**AGG**	111	25	2	1,493	18	7	3	255	4	1,570	274	**57**	328	4	1	265	3			22	[illegible]

	-	AAA	AAC	AAG	AAT	ACA	ACC	ACG	ACT	AGA	AGC	AGG	AGT	ATA	ATC	ATG	ATT	CAA	CAC	CAG	CAT
**AGT**	167	12	30	22	1,764	8	13	3	743	301	1,843	282	**95**	4	7	7	446	2			25
**ATA**	112	337	2	14	15	969	7	20	11	285	4	4	9	**68**	663	1,385	926	9	2		1
**ATC**	89	4	158	3	7	7	514	2	16	1	145	2	10	716	**57**	403	2,538		1	1	
**ATG**	252	8	13	480	41	78	10	1,362	18	12	6	305	4	1,580	416	**105**	632			9	5
**ATT**	182	12	14	3	488	11	20	10	1,214	5	9	4	388	882	2,605	604	**133**				10
**CAA**	210	747	7	13	10	12		1		18	7			5		1	1	**111**	339	2,348	425
**CAC**	79	3	218	2	8	1	3		2	2	3	1			1	1		317	**44**	378	1,590
**CAG**	180	12	4	522	4	2			1			8	1	1	1	4		2,296	368	**73**	434
**CAT**	174	6	8	2	569	7			4				19			1	6	470	1,749	377	**75**
**CCA**	258	4	3	1		292	1	5	4	9				7		1		390	6	20	8
**CCC**	111		2			2	145		8		2				3			3	127	4	4
**CCG**	84			4		4		78	2			1	1			2		6	2	246	3
**CCT**	218				14	3	4	2	383	1			5			1	2	5	10	9	292
**CGA**	45	19	2		1	1	1			524	3	11		2		1	1	1,679	6	29	12
**CGC**	28	2	5				1	1		3	159	2			3			8	771	8	20
**CGG**	40			6	1	1		4		12	2	404	2			4		25	6	1,429	19
**CGT**	47			2	12				9	11	2	5	227				3	14	17	16	1,631
**CTA**	88	3		1	1	5				4		1		304	2	9	9	284		4	2
**CTC**	117		1				4	1			3		2	3	275	5	9	6	142	3	3
**CTG**	85	1	1	3	1	2		2				2		13	2	453	6	7		342	2
**CTT**	192	1	1	3	1		1		17				4	10	6	3	656	11	4	8	335
**GAA**	393	2,391	17	46	26	14	1	5	1	42		2	1	10	2		1	830	2	10	8
**GAC**	146	17	1,050	12	30		5	1	3	1	28	1		1	3	1	2	2	217	2	9
**GAG**	291	45	19	2,019	26	1		11		8	3	41	1	2		14		22	3	860	3
**GAT**	346	18	34	12	2,287		1	1	13	4	1	2	50	1	1	2	30	2	7	9	555
**GCA**	211	29				1,830	7	29	10	10	3		1	57		6		16		1	
**GCC**	121		9	1	1	4	1,040	3	34	1	10		1		11	1	1		3	1	
**GCG**	53			16	2	27		346	7	3		5				31				4	
**GCT**	223	1	3		21	12	41	8	1,886	1	5	2	14	1			43	4		1	8
**GGA**	267	46		3	3	12		2	1	1,202	4	14	14	6	1			23	1	2	1
**GGC**	132	6	39	1	5		4			6	895	8	29		1		1		12		
**GGG**	161	1	1	33	7	1	2	4	1	10	6	730	11			8		1	2	20	
**GGT**	291	2	2	6	41				12	9	17	9	1,616		1	2	16				29
**GTA**	83	8		1	1	30				3	2		1	1,368	3	24	15	4			
**GTC**	59		1	1			16				1		1	7	1,154	8	32	3	1	1	1
**GTG**	134	3		14		5	1	29				5	3	38	7	1,392	13		1	6	1
**GTT**	201	3			11	4	4		31	1	1		22	27	27	9	2,615	1			2
**TAA**	26	34												1				68	2		
**TAC**	90	9	243	8	4		1				4	1			5			13	561	4	25
**TAG**	20			19														2		59	
**TAT**	143	3	9		453			5	3		1		8	4		1	10	8	20		1,155
**TCA**	208	12	1		2	444	7	9	4	4		2		12	3	1		7			1
**TCC**	110		9		1	2	219		11		8				3			2	3	1	
**TCG**	63			3	1	4	3	91	1	2		3				8	1		1	4	
**TCT**	346		1		20	2	15	1	493		2		14	3			6				29
**TGA**	34									16			1			1		10			
**TGC**	80		8			1	3			1	154	1	6		2	1			34	1	
**TGG**	94	1		7				1		2	3	201				9		4	1	57	
**TGT**	117		1		5	1	1		2	2	3	1	300				8	4	3	2	56
**TTA**	118	6	1		2	10	1	1	1	2				369	10	8	10	3			1
**TTC**	165	1	2		2		5			1			1	3	252	6	13	3	14		2
**TTG**	187			8	2	3		11	4			3		10		615	6	2		11	
**TTT**	292			2	6		1		16				4	5	8	5	548	2	1	1	11

Amino acid changes

How to read this table:
- Rows are reference amino acids and columns are changed amino acids. E.g. Row 'A' column 'E' indicates how many 'A' amino acids have been replaced by 'E' amino acids.
- Red background colors indicate that more changes happened (heat-map).
- Diagonals are indicated using grey background color
- WARNING: This table may include different translation codon tables (e.g. mamalian DNA and mitochondrial DNA).

	*	-	?	A	C	D	E	F	G	H	I	K	L	M	N	P	Q	R	S	T	
*****	**341**	65	15		14	1	55	4	23	2	1	53	69	1		4	139	91	62		
**-**	62		2,128	236	66	211	316	172	295	179	149	240	301	67	202	321	229	200	525	246	
**?**																					
**A**	8	577	31	**18,121**	26	612	878	27	1,744	11	113	47	69	38	36	1,276	27	32	2,059	5,284	5
**C**	99	184	13	9	**3,129**	13	3	536	380	93	10		42	1	14	20	7	1,332	1,205	8	

	*	-	?	A	C	D	E	F	G	H	I	K	L	M	N	P	Q	R	S	T	
**D**	15	470	22	560	25	**7,370**	3,344	13	1,977	788	38	59	15	3	3,401	10	15	28	101	24	
**E**	312	649	35	879	1	3,400	**6,156**	10	2,308	16	15	4,501	42	14	88	14	1,722	122	12	33	
**F**	12	423	34	24	546	20	2	**5,893**	12	28	829	3	3,809	11	10	33	6	8	1,519	22	
**G**	122	805	46	1,732	542	2,127	2,164	19	**13,757**	45	26	98	24	10	98	10	46	2,864	2,624	39	[illegible]
**H**	10	236	17	25	129	666	26	28	36	**3,458**	7	13	512	2	803	382	1,542	2,140	47	17	
**I**	5	351	32	75	6	21	11	882	31	14	**8,588**	373	1,565	2,392	684	22	10	312	592	2,801	[illegible]
**K**	227	625	43	42	2	60	4,026	3	75	21	396	**6,423**	23	563	2,818	3	1,378	3,245	66	1,004	
**L**	228	723	64	51	42	8	25	4,172	23	489	1,700	26	**27,536**	1,093	11	2,641	681	760	2,495	63	[illegible]
**M**	5	238	14	27	4	7	24	18	10	5	2,628	488	1,177	**105**	54	8	9	319	21	1,468	[illegible]
**N**	8	440	24	24	19	3,181	71	14	80	753	701	2,733	11	11	**6,334**	7	40	61	3,095	943	
**P**	16	644	27	1,254	13	8	12	27	15	452	12	9	3,013	4	19	**15,176**	683	784	3,218	933	
**Q**	746	367	23	19	3	27	1,598	4	39	1,566	8	1,294	765	5	25	646	**4,828**	2,960	32	16	
**R**	517	403	38	33	1,778	36	82	25	2,609	2,484	342	3,131	792	275	63	758	3,252	**9,727**	1,562	734	
**S**	258	938	42	2,000	1,577	71	31	1,880	2,623	66	681	86	2,800	17	3,024	2,904	17	1,505	**20,905**	2,481	
**T**	3	431	26	4,992	5	28	37	26	46	14	3,077	982	50	1,446	1,050	870	16	716	2,564	**14,994**	
**V**	1	444	33	4,700	16	442	581	1,050	1,033	6	5,306	30	3,056	1,433	13	30	15	27	66	120	[illegible]
**W**	488	87	7	4	293	1	8	14	166	1		8	303	9		6	61	851	173	1	
**Y**	363	209	24	7	1,443	664	25	1,095	24	1,761	19	20	60	1	709	6	25	36	666	9	

Variants by chromosome

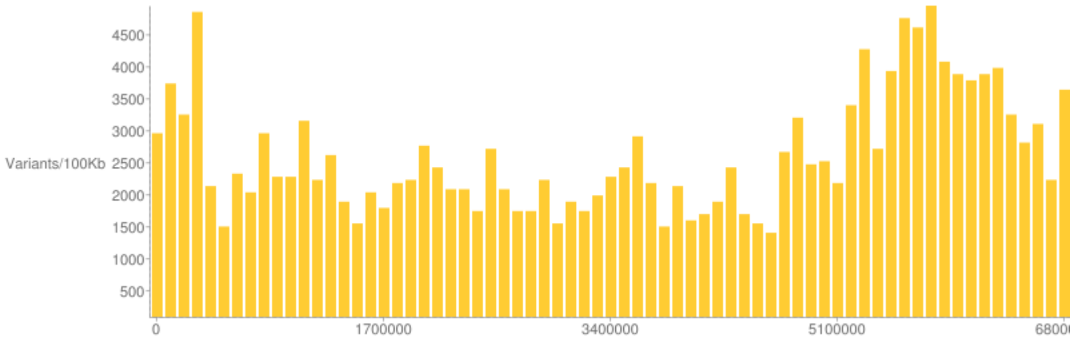

1, Position,0,100000,200000,300000,400000,500000,600000,700000,800000,900000,1000000,1100000,1200000,1300000,1400000,1500000,1600000
1,Count,2972,3768,3277,4870,2163,1506,2372,2033,2991,2322,2312,3193,2245,2622,1932,1573,2063,1827,2222,2259,2798,2454,2096,2092,1752,2732,2121,1750,

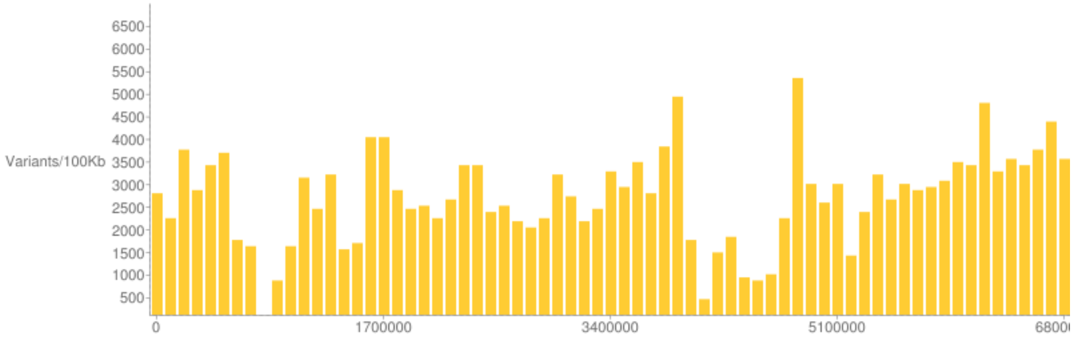

2, Position,0,100000,200000,300000,400000,500000,600000,700000,800000,900000,1000000,1100000,1200000,1300000,1400000,1500000,1600000
2,Count,2858,2251,3809,2930,3484,3730,1778,1660,113,916,1646,3175,2491,3233,1613,1742,4046,4043,2888,2482,2561,2311,2703,3468,3457,2440,2566,2190,20

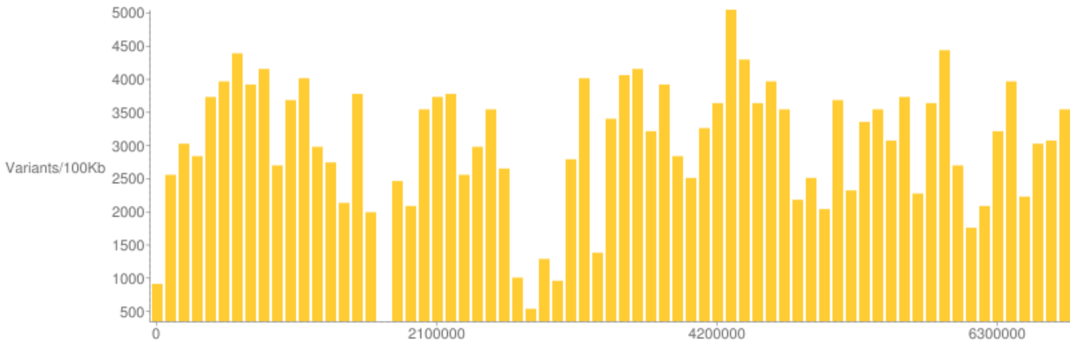

3, Position,0,100000,200000,300000,400000,500000,600000,700000,800000,900000,1000000,1100000,1200000,1300000,1400000,1500000,1600000
3,Count,920,2584,3054,2855,3757,3974,4386,3914,4193,2722,3713,4044,2999,2781,2157,3791,2015,344,2489,2080,3553,3735,3780,2559,3017,3540,2661,1016,52

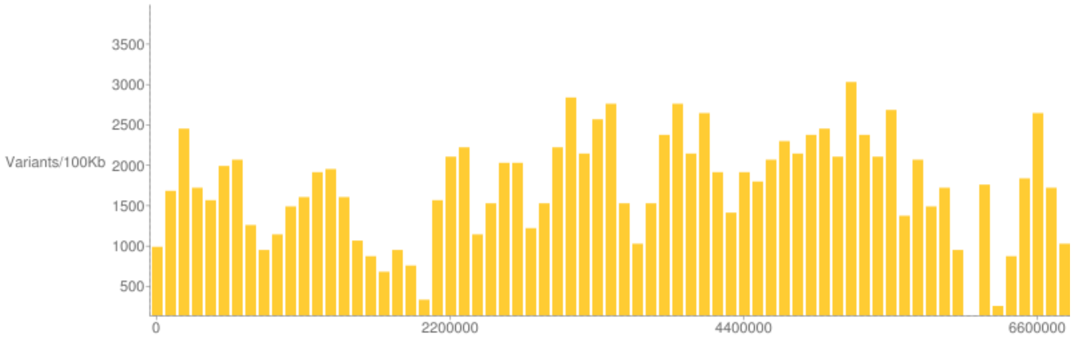

4, Position,0,100000,200000,300000,400000,500000,600000,700000,800000,900000,1000000,1100000,1200000,1300000,1400000,1500000,1600000
4,Count,1004,1677,2464,1728,1563,2017,2066,1272,970,1150,1486,1614,1939,1964,1608,1064,870,689,977,769,365,1569,2133,2233,1152,1522,2056,2043,1237,1

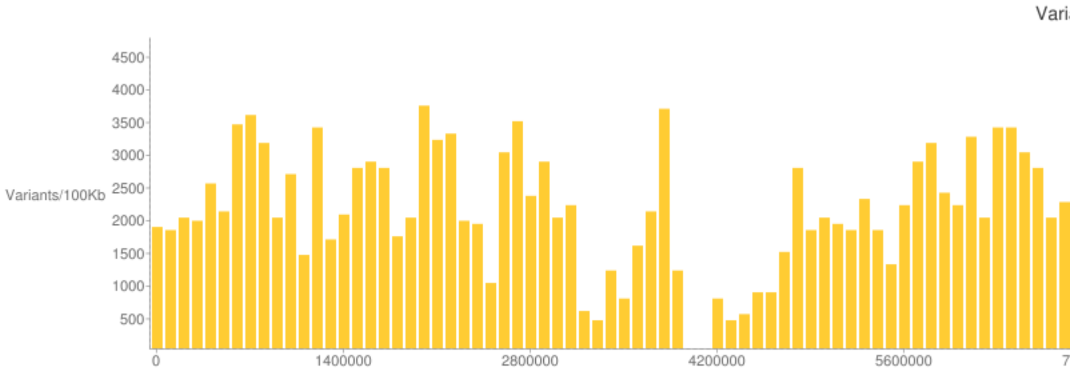

5, Position,0,100000,200000,300000,400000,500000,600000,700000,800000,900000,1000000,1100000,1200000,1300000,1400000,1500000,1600000
5,Count,1920,1869,2062,2002,2568,2135,3481,3644,3224,2064,2732,1475,3417,1745,2110,2834,2919,2830,1773,2038,3796,3260,3325,2005,1943,1062,3068,3528,

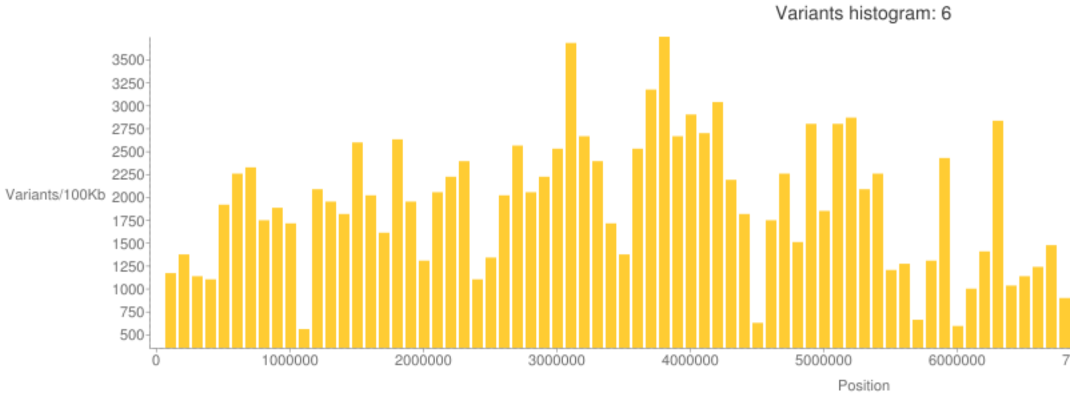

6, Position,0,100000,200000,300000,400000,500000,600000,700000,800000,900000,1000000,1100000,1200000,1300000,1400000,1500000,1600000
6,Count,370,1178,1380,1159,1130,1917,2266,2350,1750,1906,1716,563,2091,1961,1816,2612,2020,1613,2635,1968,1315,2083,2228,2400,1115,1370,2040,2582,20

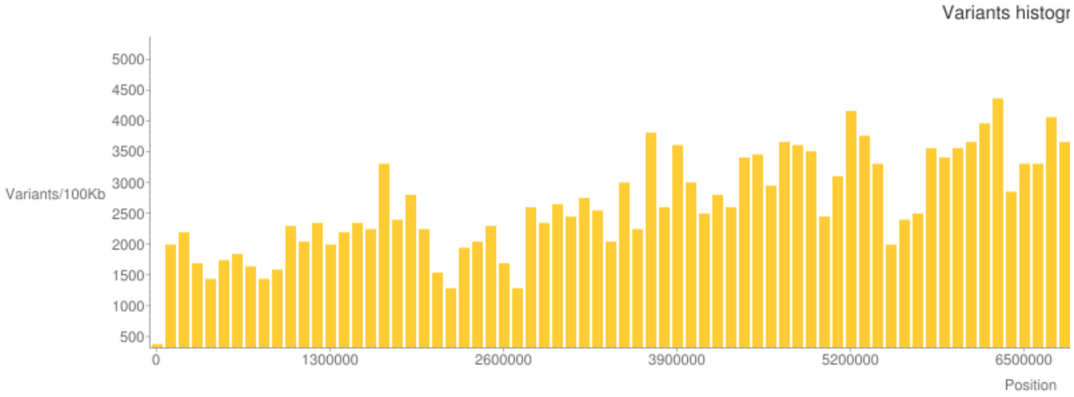

7, Position,0,100000,200000,300000,400000,500000,600000,700000,800000,900000,1000000,1100000,1200000,1300000,1400000,1500000,1600000
7,Count,397,1991,2205,1695,1427,1756,1872,1653,1448,1583,2296,2051,2347,2014,2218,2381,2279,3338,2421,2791,2278,1575,1284,1976,2059,2289,1712,1280,2

8, Position,0,100000,200000,300000,400000,500000,600000,700000,800000,900000,1000000,1100000,1200000,1300000,1400000,1500000,1600000
8,Count,2682,1642,2886,2396,1649,2230,2391,1835,2043,1594,2688,2463,1610,1663,2039,1310,1162,1238,1400,2105,3511,4924,4353,4943,3199,4473,4283,4478,

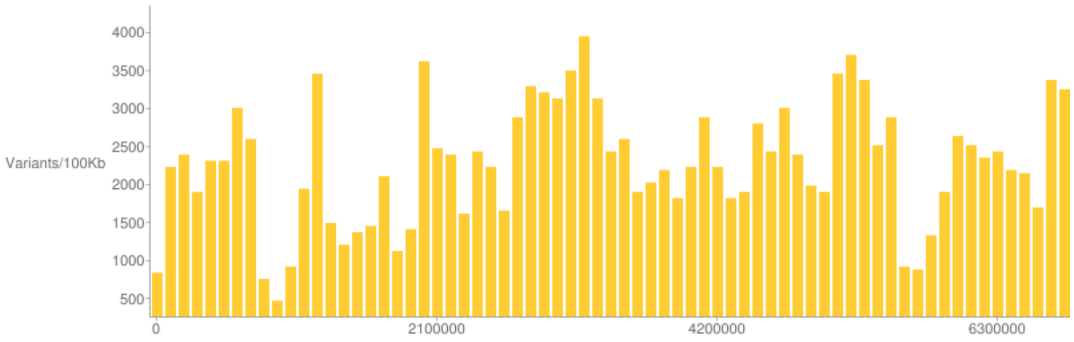

9, Position,0,100000,200000,300000,400000,500000,600000,700000,800000,900000,1000000,1100000,1200000,1300000,1400000,1500000,1600000
9,Count,838,2233,2405,1919,2307,2311,3021,2614,759,472,914,1950,3481,1487,1218,1388,1486,2141,1143,1410,3639,2476,2401,1613,2435,2248,1651,2879,3288

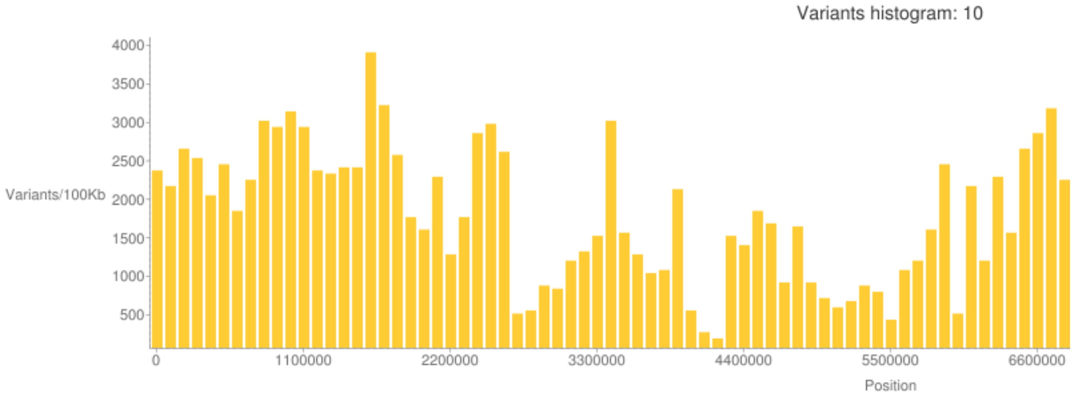


```
10, Position,0,100000,200000,300000,400000,500000,600000,700000,800000,900000,1000000,1100000,1200000,1300000,1400000,1500000,160000
10,Count,2372,2202,2651,2558,2079,2485,1881,2272,3016,2944,3156,2969,2369,2350,2418,2431,3921,3244,2589,1797,1620,2305,1278,1779,2884,2992,2630,510,
```

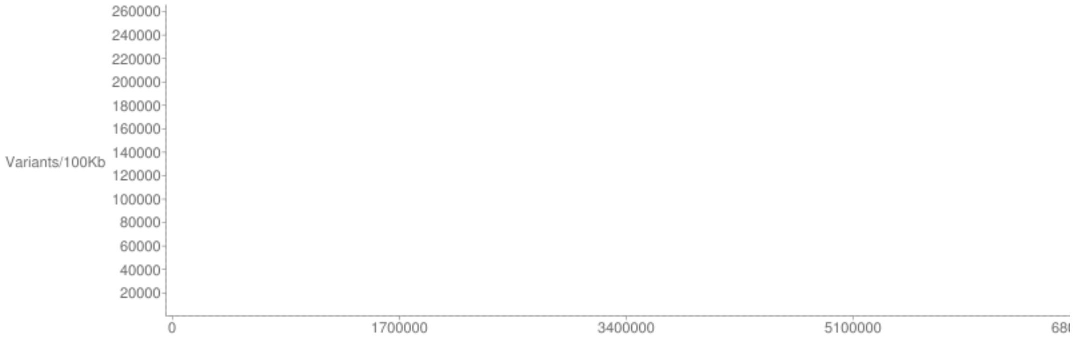


```
11, Position,0,100000,200000,300000,400000,500000,600000,700000,800000,900000,1000000,1100000,1200000,1300000,1400000,1500000,160000
11,Count,2313,1910,1794,2412,2066,2072,1895,2009,1960,1611,1380,1884,1704,1165,1764,2015,2367,2238,2045,1606,2685,2146,2170,2173,2465,2467,1793,2440
```

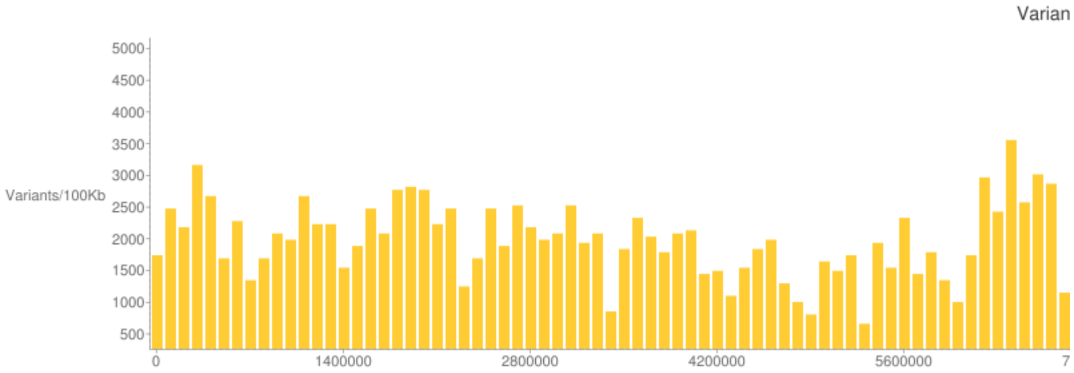


```
12, Position,0,100000,200000,300000,400000,500000,600000,700000,800000,900000,1000000,1100000,1200000,1300000,1400000,1500000,160000
12,Count,1770,2503,2206,3202,2701,1688,2299,1379,1712,2119,2021,2664,2227,2224,1569,1916,2515,2102,2785,2836,2798,2244,2486,1284,1707,2490,1922,2555
```

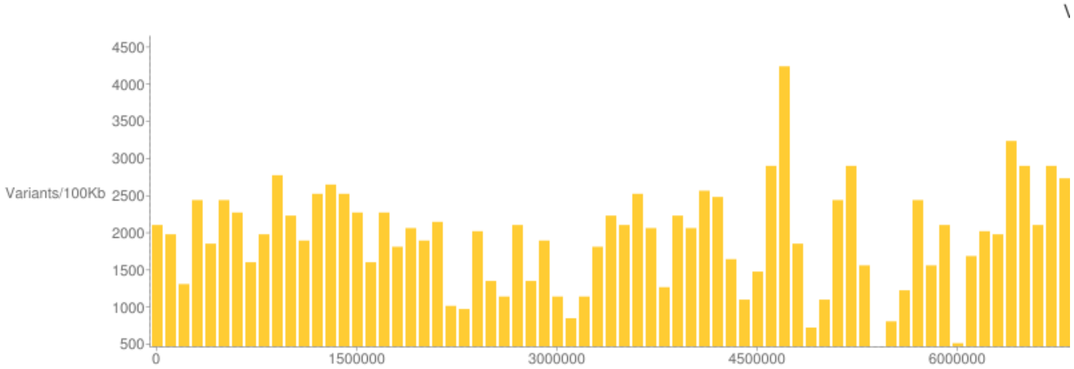

13, Position,0,100000,200000,300000,400000,500000,600000,700000,800000,900000,1000000,1100000,1200000,1300000,1400000,1500000,160000
13,Count,2124,1996,1336,2449,1885,2455,2265,1624,1980,2803,2229,1888,2541,2673,2556,2285,1636,2292,1811,2085,1929,2162,1050,1001,2051,1345,1151,2134

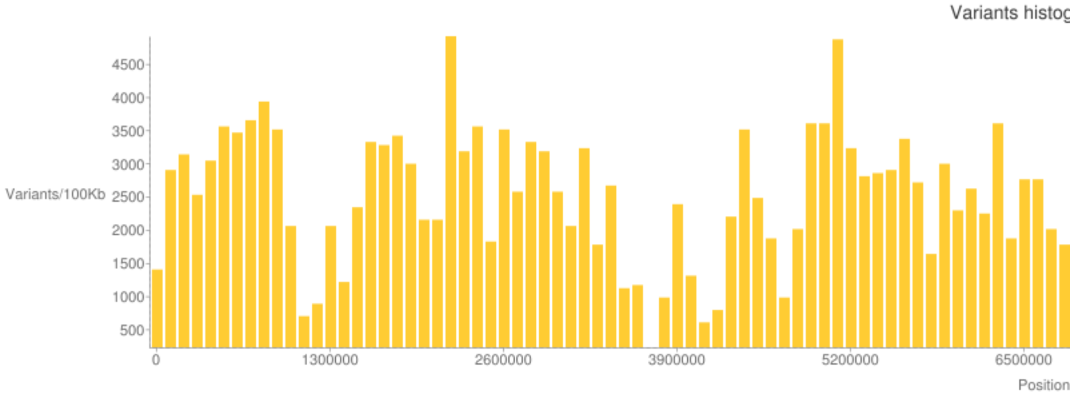

14, Position,0,100000,200000,300000,400000,500000,600000,700000,800000,900000,1000000,1100000,1200000,1300000,1400000,1500000,160000
14,Count,1417,2906,3153,2532,3046,3590,3489,3682,3935,3543,2068,722,887,2075,1249,2345,3323,3303,3429,3003,2171,2159,4915,3185,3581,1843,3548,2615,3

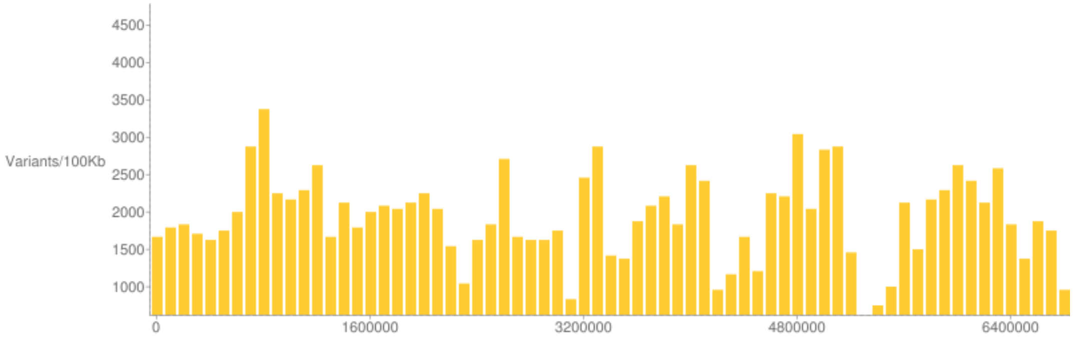

15, Position,0,100000,200000,300000,400000,500000,600000,700000,800000,900000,1000000,1100000,1200000,1300000,1400000,1500000,160000
15,Count,1689,1809,1831,1716,1640,1760,1998,2892,3381,2249,2194,2319,2640,1669,2137,1811,2020,2096,2046,2127,2256,2049,1565,1068,1644,1863,2727,1672

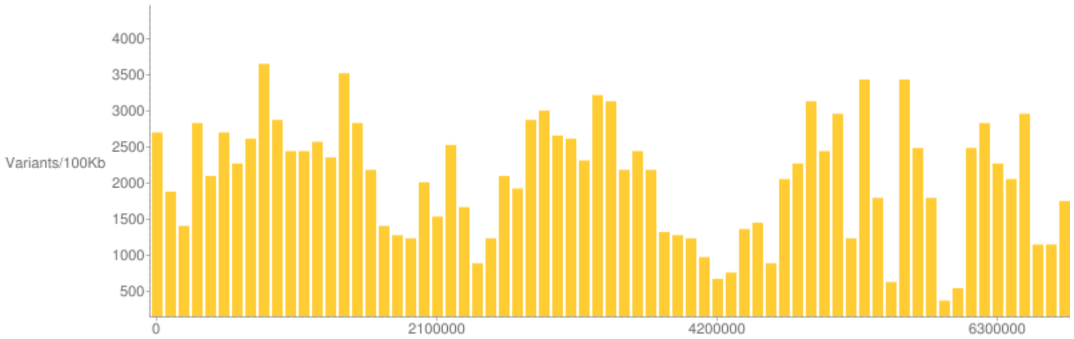


```
16, Position,0,100000,200000,300000,400000,500000,600000,700000,800000,900000,1000000,1100000,1200000,1300000,1400000,1500000,160000
16,Count,2700,1892,1435,2859,2092,2696,2289,2610,3658,2867,2447,2449,2582,2357,3520,2859,2215,1434,1301,1250,2016,1552,2548,1661,889,1249,2094,1934,
```

```
17, Position,0,100000,200000,300000,400000,500000,600000,700000,800000,900000,1000000,1100000,1200000,1300000,1400000,1500000,160000
17,Count,2148,1285,2317,2320,1542,1713,2329,2272,2284,1588,2333,3296,2700,3151,3303,2914,2837,1287,1495,3364,2650,2669,2756,2712,2531,4535,3230,3253
```

```
18, Position,0,100000,200000,300000,400000,500000,600000,700000,800000,900000,1000000,1100000,1200000,1300000,1400000,1500000,160000
18,Count,1099,1107,1631,187,626,1766,1266,559,2519,2365,3083,3136,3698,2255,2254,1613,2339,2442,2145,2622,2737,2774,2907,2762,2080,2333,3232,2033,23
```

```
19, Position,0,100000,200000,300000,400000,500000,600000,700000,800000,900000,1000000,1100000,1200000,1300000,1400000,1500000,160000
19,Count,2023,1647,1979,2202,2177,1629,2163,2302,1891,2360,2617,2591,2153,2464,2191,1884,2780,2744,2204,2035,1657,2280,263972,2539,2403,2213,2153,22
```

```
20, Position,0,100000,200000,300000,400000,500000,600000,700000,800000,900000,1000000,1100000,1200000,1300000,1400000,1500000,160000
20,Count,2740,2855,1994,2438,1725,2149,1279,1755,428,1976,2140,1497,1643,2556,2223,574,241,471,2528,2440,2496,2557,2289,2831,2090,2120,930,828,1889,
```

```
10000001, Position,0,1000000,2000000,3000000,4000000,5000000,6000000,7000000,8000000,9000000,10000000,11000000,12000000,13000000,140
10000001,Count,23920,19793,18295,15749,26687,14554,12893,19072,6368,13221,9353,284,218,136,1933,206,214,10281,12741,10913,5814,258,180,2337,21461,18
```

Details by gene

Here you can find a tab-separated table.
