# Supplementary material for: The ‘Tommy Atkins’ mango genome reveals candidate genes for fruit quality
Source: BMC Plant Biol. 2021 Feb 22;21:108. doi: 10.1186/s12870-021-02858-1 (PMC7898432; doi:10.1186/s12870-021-02858-1)
Supplement: Supplementary file 3 — Additional file 3: Supplemental Data S3. Fruit weight LG4 QTL report. Supplemental Data S4. Fruit weight LG7 QTL report. Supplemental Table 1. Summary of the M. indica genome assembly by chromosome. Supplemental Table 2. BUSCO analysis for the consensus diploid genome assembly TA4. Supplemental Table 3. Occurrence and distribution of repetitive DNA sequences in the mango genome. Supplemental Table 4. Protein domain content. Supplemental Table 5. Number of homologs pairs with Ks between 0.1 and 0.6 per chromosome. Supplemental Table 6. Repetitive elements comparative analysis. Supplemental Table 7. Comparison of the SNP variants and variants rate for ‘Tommy Atkins’ and ‘Kensington Pride’ by pseudomolecule. Supplemental Table S8. Concurrence of linkage groups between ‘Tommy Atkins’ and ‘Alphonso’. Supplementary Figure 1. Repeat composition for M. indica, P. vera and C. sinensis [file 12870_2021_2858_MOESM3_ESM.docx]

Supplemental Table 1. Summary of the *M. indica* genome assembly version TA4 by chromosome

| Chromosome | Sequence length (bp) | Gap size (bp) | Number of gaps | % of N’s |
| --- | --- | --- | --- | --- |
| 1 | 17,320,008 | 332,990 | 656 | 1.92 |
| 2 | 17,063,873 | 412,663 | 761 | 2.42 |
| 3 | 21,566,805 | 446,068 | 849 | 2.07 |
| 4 | 22,357,487 | 404,669 | 798 | 1.81 |
| 5 | 14,540,018 | 302,711 | 629 | 2.08 |
| 6 | 10,680,009 | 202,353 | 416 | 1.89 |
| 7 | 13,133,232 | 273,938 | 537 | 2.08 |
| 8 | 14,750,018 | 295,338 | 575 | 2.00 |
| 9 | 21,055,410 | 442,502 | 850 | 2.10 |
| 10 | 11,063,414 | 215,904 | 404 | 1.95 |
| 11 | 17,675,019 | 277,708 | 580 | 1.57 |
| 12 | 14,336,529 | 287,961 | 594 | 2.01 |
| 13 | 15,099,493 | 316,724 | 615 | 2.10 |
| 14 | 13,335,999 | 295,532 | 575 | 2.22 |
| 15 | 16,178,320 | 274,746 | 526 | 1.70 |
| 16 | 21,434,198 | 447,993 | 829 | 2.09 |
| 17 | 11,746,059 | 209,647 | 427 | 1.78 |
| 18 | 16,863,820 | 324,619 | 646 | 1.92 |
| 19 | 22,398,858 | 448,155 | 878 | 2.00 |
| 20 | 16,105,987 | 316,777 | 581 | 1.97 |
| Unanchored | 48,585,777 | 2,449,365 | 1,845 | 5.04 |
| TOTAL | 377,290,333 | 8,978,363 | 14,571 | 2.38 |

Supplemental Table 2. BUSCO analysis for the consensus diploid genome assembly TA4 and its proteins derived from the genome annotation using the Embryophyta 10 dataset. Shown are complete BUSCO, single copy, duplicated, fragmented, missing, and total BUSCO genes for the TA4 assembly.

| **BUSCO statistics** | **Genome Assembly TA4** | **Protein Dataset TA4** |
| --- | --- | --- |
| Complete BUSCOs | 1339 (97.4%) | 1301 (94.6%) |
| Complete BUSCOs -Single-Copy | 1166 (84.8%) | 1058 (76.9%) |
| Complete BUSCOS -Duplicated | 173 (12.6%) | 243 (17.7%) |
| Fragmented BUSCOs | 6 (0.4%) | 23 (1.7%) |
| Missing BUSCOs | 30 (2.2%) | 51 (3.7%) |
| Total BUSCO groups searched | 1375 | 1375 |

Supplemental Table 3. Occurrence and distribution of repetitive DNA sequences in the mango genome. Element is the class of repeat, Number of elements is the number of repeats detected, Length occupied is the total length of the repeat elements, and Percentage of sequence is the overall percentage of the genome represented by the specific classes of repeats.

| **Element** | **Number of elements** | **Length occupied** | **Percentage of sequence** |
| --- | --- | --- | --- |
| SINEs | 29 | 2,823 | 0.00 |
| LINEs | 3,048 | 3,442,297 | 0.91 |
| LINEs LINE1 | 2,340 | 2,804,157 | 0.74 |
| LINEs LINE2 | 708 | 638,140 | 0.17 |
| LTR elements | 69,265 | 63,590,076 | 16.85 |
| LTR elements ERV_classI | 93 | 65,788 | 0.02 |
| DNA elements | 31,960 | 17,275,845 | 4.58 |
| DNA elements hAT-Charlie | 3 | 346 | 0.00 |
| Unclassified | 228,770 | 58,748,319 | 15.57 |
| Total interspersed repeats |  | 143,059,360 | 37.92 |
| Small RNA | 110 | 115,192 | 0.03 |
| Simple repeats | 93,348 | 4,917,453 | 1.3 |
| Low complexity | 18,476 | 931,343 | 0.25 |

Supplemental Table 4. Protein domain content. Shown are multiple classes of protein domains and the number of annotated genes with identified protein domains.

| **Protein Domain** | **Count** |
| --- | --- |
| PPR repeat | 1722 |
| PPR repeat family | 1259 |
| Leucine Rich Repeat | 1185 |
| Protein kinase domain | 1119 |
| WD domain, G-beta repeat | 980 |
| Leucine rich repeat | 825 |
| Myb-like DNA-binding domain | 486 |
| Cytochrome P450 | 468 |
| Protein tyrosine kinase | 460 |
| NB-ARC domain | 455 |
| RNA recognition motif. (a.k.a. RRM, RBD, or RNP domain) | 443 |
| Leucine rich repeat N-terminal domain | 330 |
| Leucine Rich repeat | 304 |
| Ankyrin repeats (3 copies) | 283 |
| Leucine Rich repeats (2 copies) | 267 |
| Mitochondrial carrier protein | 256 |
| Multicopper oxidase | 232 |
| EF-hand domain pair | 208 |
| Tetratricopeptide repeat | 208 |
| ABC transporter | 207 |
| Ring finger domain | 199 |
| AP2 domain | 177 |

Supplemental Table 5. Number of homologs pairs with Ks between 0.1 and 0.6 per chromosome. Relations of the unanchored scaffolds are noted as U. Relations with more than 100 pairs have been black shaded.

|  | **1** | **2** | **3** | **4** | **5** | **6** | **7** | **8** | **9** | **10** | **11** | **12** | **13** | **14** | **15** | **16** | **17** | **18** | **19** | **20** | **U** |
| --- | --- | --- | --- | --- | --- | --- | --- | --- | --- | --- | --- | --- | --- | --- | --- | --- | --- | --- | --- | --- | --- |
| **1** | 62 | 4 | 294 | 6 | 5 | 2 | 5 | 10 | 6 | 22 | 9 | 7 | 18 | 8 | 5 | 5 | 7 | 12 | 9 | 337 | 9 |
| **2** |  | 70 | 232 | 186 | 6 | 12 | 5 | 3 | 6 | 4 | 8 | 58 | 15 | 7 | 6 | 3 | 2 | 15 | 2 | 3 | 70 |
| **3** |  |  | 217 | 1 | 3 | 10 | 6 | 4 | 10 | 7 | 8 | 17 | 14 | 5 | 7 | 20 | 5 | 6 | 15 | 10 | 60 |
| **4** |  |  |  | 88 | 3 | 8 | 7 | 7 | 13 | 10 | 58 | 35 | 8 | 9 | 3 | 21 | 325 | 244 | 7 | 28 | 13 |
| **5** |  |  |  |  | 65 | 3 | 439 | 4 | 5 | 8 | 3 | 1 | 5 | 3 | 1 | 6 | 0 | 3 | 5 | 4 | 58 |
| **6** |  |  |  |  |  | 37 | 2 | 7 | 4 | 6 | 7 | 7 | 5 | 7 | 3 | 22 | 4 | 12 | 230 | 3 | 17 |
| **7** |  |  |  |  |  |  | 84 | 6 | 8 | 4 | 2 | 45 | 5 | 6 | 1 | 4 | 8 | 6 | 8 | 1 | 17 |
| **8** |  |  |  |  |  |  |  | 78 | 52 | 2 | 8 | 11 | 197 | 3 | 0 | 7 | 2 | 192 | 6 | 1 | 111 |
| **9** |  |  |  |  |  |  |  |  | 43 | 3 | 16 | 17 | 222 | 11 | 10 | 319 | 5 | 6 | 7 | 2 | 58 |
| **10** |  |  |  |  |  |  |  |  |  | 66 | 14 | 18 | 7 | 7 | 5 | 20 | 8 | 11 | 402 | 21 | 35 |
| **11** |  |  |  |  |  |  |  |  |  |  | 31 | 317 | 4 | 8 | 4 | 509 | 2 | 4 | 8 | 7 | 17 |
| **12** |  |  |  |  |  |  |  |  |  |  |  | 180 | 2 | 8 | 8 | 10 | 57 | 4 | 3 | 8 | 16 |
| **13** |  |  |  |  |  |  |  |  |  |  |  |  | 47 | 7 | 4 | 39 | 5 | 52 | 17 | 4 | 52 |
| **14** |  |  |  |  |  |  |  |  |  |  |  |  |  | 37 | 518 | 9 | 4 | 6 | 10 | 7 | 23 |
| **15** |  |  |  |  |  |  |  |  |  |  |  |  |  |  | 33 | 9 | 3 | 6 | 13 | 7 | 17 |
| **16** |  |  |  |  |  |  |  |  |  |  |  |  |  |  |  | 66 | 4 | 10 | 18 | 7 | 17 |
| **17** |  |  |  |  |  |  |  |  |  |  |  |  |  |  |  |  | 50 | 5 | 5 | 3 | 50 |
| **18** |  |  |  |  |  |  |  |  |  |  |  |  |  |  |  |  |  | 182 | 35 | 3 | 46 |
| **19** |  |  |  |  |  |  |  |  |  |  |  |  |  |  |  |  |  |  | 52 | 12 | 81 |
| **20** |  |  |  |  |  |  |  |  |  |  |  |  |  |  |  |  |  |  |  | 75 | 27 |
| **U** |  |  |  |  |  |  |  |  |  |  |  |  |  |  |  |  |  |  |  |  | 128 |

Supplemental Table 6. Repetitive elements comparative analysis.

|  | M_indica | | | C_sinensis | | | A_occidentale | | | P_vera | | |
| --- | --- | --- | --- | --- | --- | --- | --- | --- | --- | --- | --- | --- |
| TE Family | number | size (bp) | portion (%) | number | size (bp) | portion (%) | number | size (bp) | portion (%) | number | size (bp) | portion (%) |
| All | 408786 | 181373851 | 48.07% | 267925 | 124103670 | 38.88% | 347694 | 221220056 | 49.91% | 536090 | 440090475 | 65.56% |
| Class I | 260685 | 128898479 | 34.16% | 164022 | 94366784 | 29.56% | 268784 | 190433893 | 42.97% | 412386 | 368727538 | 54.93% |
| LTR | 182807 | 104243590 | 27.63% | 142595 | 84484876 | 26.47% | 213730 | 166028764 | 37.46% | 363391 | 348346961 | 51.89% |
| LTR/Copia | 75587 | 50596306 | 13.41% | 59192 | 32683278 | 10.24% | 65869 | 49644916 | 11.20% | 143244 | 132666134 | 19.76% |
| LTR/Caulimovirus | 1197 | 920580 | 0.24% | 8043 | 6732595 | 2.11% | 652 | 572565 | 0.13% | 3940 | 3522932 | 0.52% |
| LTR/Gypsy | 105045 | 52338939 | 13.87% | 75086 | 44900930 | 14.07% | 147209 | 115811283 | 26.13% | 215860 | 211903785 | 31.57% |
| LTR/ltr_Others | 978 | 387765 | 0.10% | 274 | 168073 | 0.05% | 0 | 0 | 0.00% | 347 | 254110 | 0.04% |
| nLTR | 77878 | 24654889 | 6.53% | 21427 | 9881908 | 3.10% | 55054 | 24405129 | 5.51% | 48995 | 20380577 | 3.04% |
| nLTR/LINE | 67581 | 22643726 | 6.00% | 17287 | 9001003 | 2.82% | 54126 | 24121322 | 5.44% | 34619 | 17603819 | 2.62% |
| nLTR/SINE | 7588 | 1037416 | 0.27% | 3187 | 431932 | 0.14% | 718 | 181013 | 0.04% | 12339 | 1883911 | 0.28% |
| nLTR/PLE | 2709 | 973747 | 0.26% | 953 | 448973 | 0.14% | 210 | 102794 | 0.02% | 2037 | 892847 | 0.13% |
|  |  |  |  |  |  |  |  |  |  |  |  |  |
| ClassII | 127041 | 45931312 | 12.17% | 101526 | 28581230 | 8.95% | 72896 | 28075150 | 6.33% | 112246 | 64027451 | 9.54% |
| DNA_MITE | 81560 | 19747235 | 5.23% | 62957 | 13460651 | 4.22% | 36657 | 8960865 | 2.02% | 27486 | 7370232 | 1.10% |
| DNA_MITE/CACTA | 4345 | 857791 | 0.23% | 3166 | 601642 | 0.19% | 11271 | 2772604 | 0.63% | 1203 | 340855 | 0.05% |
| DNA_MITE/Harbinger | 2846 | 673129 | 0.18% | 10434 | 1977764 | 0.62% | 3901 | 1053784 | 0.24% | 1323 | 276475 | 0.04% |
| DNA_MITE/hAT | 35284 | 8766542 | 2.32% | 16265 | 4144945 | 1.30% | 10561 | 2589562 | 0.58% | 12159 | 3539855 | 0.53% |
| DNA_MITE/mite_Others | 506 | 101572 | 0.03% | 609 | 77387 | 0.02% | 2 | 442 | 0.00% | 0 | 0 | 0.00% |
| DNA_MITE/MuDR | 0 | 0 | 0.00% | 1697 | 429064 | 0.13% | 0 | 0 | 0.00% | 292 | 42878 | 0.01% |
| DNA_MITE/Mutator | 12101 | 3324267 | 0.88% | 10296 | 2206108 | 0.69% | 4397 | 1282663 | 0.29% | 5990 | 1479804 | 0.22% |
| DNA_MITE/Tc | 26478 | 6023934 | 1.60% | 20490 | 4023741 | 1.26% | 6525 | 1261810 | 0.28% | 6519 | 1690365 | 0.25% |
| DNA_nMITE | 45481 | 26184077 | 6.94% | 38569 | 15120579 | 4.74% | 36239 | 19114285 | 4.31% | 84760 | 56657219 | 8.44% |
| DNA_nMITE/CACTA | 1258 | 747071 | 0.20% | 3491 | 2094920 | 0.66% | 10953 | 6765019 | 1.53% | 6775 | 3640747 | 0.54% |
| DNA_nMITE/Harbinger | 2202 | 899520 | 0.24% | 2545 | 908046 | 0.28% | 371 | 165966 | 0.04% | 11022 | 5849062 | 0.87% |
| DNA_nMITE/hAT | 11463 | 5117929 | 1.36% | 14826 | 4965602 | 1.56% | 7407 | 3105818 | 0.70% | 35197 | 22703248 | 3.38% |
| DNA_nMITE/MuDR | 18405 | 14608080 | 3.87% | 7301 | 3695562 | 1.16% | 5006 | 2511945 | 0.57% | 10949 | 9567150 | 1.43% |
| DNA_nMITE/Mutator | 3297 | 1244863 | 0.33% | 2586 | 1063526 | 0.33% | 6625 | 3179152 | 0.72% | 10325 | 9837078 | 1.47% |
| DNA_nMITE/Tc | 8856 | 3566614 | 0.95% | 7820 | 2392923 | 0.75% | 5877 | 3386385 | 0.76% | 10492 | 5059934 | 0.75% |
|  |  |  |  |  |  |  |  |  |  |  |  |  |
| Helitron | 21060 | 6544060 | 1.73% | 2377 | 1155656 | 0.36% | 6014 | 2711013 | 0.61% | 11458 | 7335486 | 1.09% |

Supplemental Table 7. Comparison of the SNP variants and variants rate for ‘Tommy Atkins’ and ‘Kensington Pride’ by pseudomolecule. Pseudomolecule 10000001 includes the unmapped reads from the TA4 assembly. Length is the length of the pseudomolecule, Variants is the number of identified variants, Variants rate is the average occurrence of a variant in base pairs.

|  |  | **Tommy Atkins** | | **Kensington Pride** | |
| --- | --- | --- | --- | --- | --- |
| **Pseudomolecule** | **Length** | **Variants** | **Variants rate** | **Variants** | **Variants rate** |
| 1 | 17,320,008 | 247,141 | 70 | 466,510 | 37 |
| 2 | 17,063,873 | 260,967 | 65 | 428,844 | 39 |
| 3 | 21,566,805 | 288,912 | 74 | 530,741 | 40 |
| 4 | 22,357,487 | 255,999 | 87 | 484,241 | 46 |
| 5 | 14,540,018 | 227,490 | 63 | 324,800 | 44 |
| 6 | 10,680,009 | 134,363 | 79 | 206,656 | 51 |
| 7 | 13,133,232 | 218,581 | 60 | 361,416 | 36 |
| 8 | 14,750,018 | 223,076 | 66 | 356,138 | 41 |
| 9 | 21,055,410 | 317,903 | 66 | 481,402 | 43 |
| 10 | 11,063,414 | 146,226 | 75 | 222,749 | 49 |
| 11 | 17,675,019 | 197,115 | 89 | 690,820 | 25 |
| 12 | 14,336,529 | 227,439 | 63 | 310,197 | 46 |
| 13 | 15,099,493 | 205,623 | 73 | 357,215 | 42 |
| 14 | 13,335,999 | 208,487 | 63 | 328,449 | 40 |
| 15 | 16,178,320 | 219,588 | 73 | 329,319 | 49 |
| 16 | 21,434,198 | 329,642 | 65 | 430,451 | 49 |
| 17 | 11,746,059 | 157,361 | 74 | 324,153 | 36 |
| 18 | 16,863,820 | 231,421 | 72 | 401,989 | 41 |
| 19 | 22,398,858 | 349,731 | 64 | 800,148 | 27 |
| 20 | 16,105,987 | 443,146 | 36 | 621,593 | 25 |
| Unanchored | 48,585,777 | 504,745 | 96 | 572,311 | 84 |
| Total | 377,290,333 | 5,394,956 | 69 | 9,030,142 | 41 |


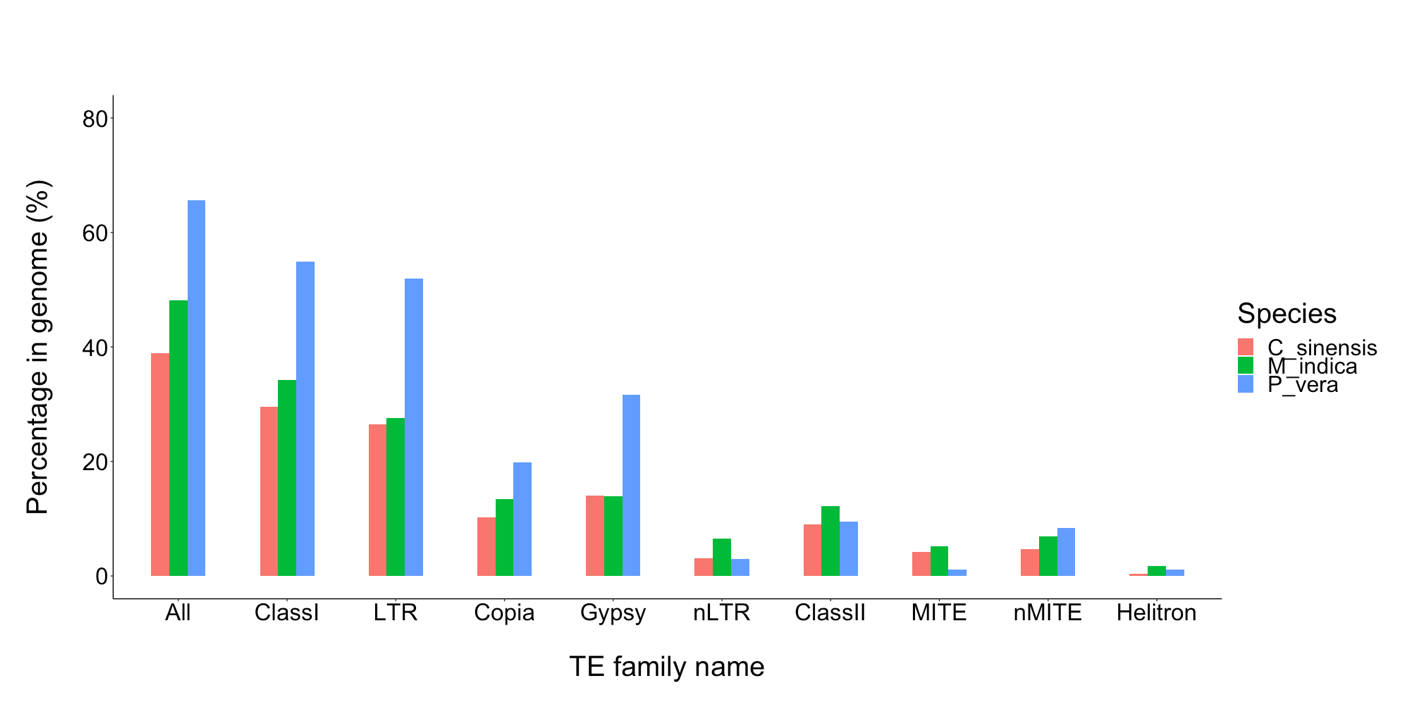


Supplementary Figure 1. Repeat composition for *M. indica, P. vera* and *C. sinensis*.

Supplemental Data 3

ANOVA and post hoc Tukey multiple comparison of means of fruit weight for LG4 QTL region

Four groups for ANOVA are: 1. individuals with haplotype TA1KP1; 2. Individuals with haplotype TA1KP2; 3. Individuals with haplotype TA2KP1; 4. Individuals with haplotype TA2KP2

Groups TA1KP1 TA1KP2 TA2KP1 TA2xKP2

Fruit weight means for groups 367.8500 426.8040 329.5500 499.3111

Number of individuals in groups 12 25 14 18

ANOVA results

Df Sum Sq Mean Sq F value Pr(>F)

data1$Genotype1 3 325172 108391 4.732 0.00445 **

Residuals 75 1717980 22906

---

Signif. codes: 0 ‘***’ 0.001 ‘**’ 0.01 ‘*’ 0.05 ‘.’ 0.1 ‘ ’ 1

Tukey multiple comparisons of means

95% family-wise confidence level

Fit: aov(formula = data1$FrtWt ~ data1$Genotype1)

$`data1$Genotype1`

diff lwr upr p adj

TA1KP2-TA1KP1 58.95400 -80.70680 198.61480 0.6850516

TA2KP1-TA1KP1 -38.30000 -178.90129 102.30129 0.8905422

TA2xKP2-TA1KP1 131.46111 -16.74566 279.66788 0.1002094

TA2KP1-TA1KP2 -97.25400 -210.90067 16.39267 0.1197370

TA2xKP2-TA1KP2 72.50711 -50.42413 195.43835 0.4134336

TA2xKP2-TA2KP1 169.76111 45.76243 293.75979 0.0031550 **

Supplemental Data 4

ANOVA and post hoc Tukey multiple comparison of means of fruit weight for LG7 QTL region

Four groups for ANOVA are: 1. individuals with haplotype TA1KP1; 2. Individuals with haplotype TA1KP2; 3. Individuals with haplotype TA2KP1; 4. Individuals with haplotype TA2KP2

Groups TA1KP1 TA1KP2 TA2KP1 TA2KP2

Fruit weight means (g) 353.0 359.6 450.1 468.2

Number of individuals 16 24 24 17

ANOVA results

Df Sum Sq Mean Sq F value Pr(>F)

data1$Genotype2 3 208634 69545 2.89 0.041 *

Residuals 77 1851684 24048

---

Signif. codes: 0 ‘***’ 0.001 ‘**’ 0.01 ‘*’ 0.05 ‘.’ 0.1 ‘ ’ 1

Tukey multiple comparisons of means

95% family-wise confidence level

Fit: aov(formula = data1$FrtWt ~ data1$Genotype2)

$`data1$Genotype2`

diff lwr upr p adj

TA1KP2-TA1KP1 6.68125 -124.75130 138.1138 0.9991433

TA2KP1-TA1KP1 97.13125 -34.30130 228.5638 0.2198666

TA2KP2-TA1KP1 115.19596 -26.64807 257.0400 0.1520047

TA2KP1-TA1KP2 90.45000 -27.10684 208.0068 0.1894507

TA2KP2-TA1KP2 108.51471 -20.57761 237.6070 0.1303465

TA2KP2-TA2KP1 18.06471 -111.02761 147.1570 0.9829199

Supplemental Table S8. Comparison of the SNP variants and variants per base pair for ‘Tommy Atkins’ and ‘Kensington Pride’ by Pseudomolecule. Pseudomolecule 99 includes the unmapped reads from the TA4 assembly. Values for Alphonso are from Wang et al. 2020 and show corresponding pseudomolecule (Ps) number and length in Mb.

|  |  | **Alphonso** |  | **Tommy Atkins** | | **Kensington Pride** | |
| --- | --- | --- | --- | --- | --- | --- | --- |
| **Ps** | **Length** | **Ps** | **Mb** | **Variants** | **Per Bp** | **Variants** | **Per Bp** |
| 1 | 17,320,008 | 9 | 18 | 247,141 | 70 | 466,510 | 37 |
| 2 | 17,063,873 | 7 | 20 | 260,967 | 65 | 428,844 | 39 |
| 3 | 21,566,805 | 2 | 24 | 288,912 | 74 | 530,741 | 40 |
| 4 | 22,357,487 | 3 | 23 | 255,999 | 87 | 484,241 | 46 |
| 5 | 14,540,018 | 5 | 15 | 227,490 | 63 | 324,800 | 44 |
| 6 | 10,680,009 | 16 | 13 | 134,363 | 79 | 206,656 | 51 |
| 7 | 13,133,232 | 17 | 13 | 218,581 | 60 | 361,416 | 36 |
| 8 | 14,750,018 | 12 | 16 | 223,076 | 66 | 356,138 | 41 |
| 9 | 21,055,410 | 9 | 21 | 317,903 | 66 | 481,402 | 43 |
| 10 | 11,063,414 | 18 | 13 | 146,226 | 75 | 222,749 | 49 |
| 11 | 17,675,019 | 8 | 18 | 197,115 | 89 | 690,820 | 25 |
| 12 | 14,336,529 | 14 | 14 | 227,439 | 63 | 310,197 | 46 |
| 13 | 15,099,493 | 10 | 17 | 205,623 | 73 | 357,215 | 42 |
| 14 | 13,335,999 | 19 | 13 | 208,487 | 63 | 328,449 | 40 |
| 15 | 16,178,320 | 11 | 17 | 219,588 | 73 | 329,319 | 49 |
| 16 | 21,434,198 | 5 | 21 | 329,642 | 65 | 430,451 | 49 |
| 17 | 11,746,059 | 20 | 12 | 157,361 | 74 | 324,153 | 36 |
| 18 | 16,863,820 | 6 | 18 | 231,421 | 72 | 401,989 | 41 |
| 19 | 22,398,858 | 1 | 29 | 349,731 | 64 | 800,148 | 27 |
| 20 | 16,105,987 | 15 | 14 | 443,146 | 36 | 621,593 | 25 |
| 99 | 48,585,777 |  |  | 504,745 | 96 | 572,311 | 84 |
| Total | 377,290,333 |  |  | 5,394,956 | 69 | 9,030,142 | 41 |
